# Assessment of Alzheimer-related pathologies of dementia using machine learning feature selection

**DOI:** 10.1186/s13195-023-01195-9

**Published:** 2023-03-10

**Authors:** Mohammed D. Rajab, Emmanuel Jammeh, Teruka Taketa, Carol Brayne, Fiona E. Matthews, Li Su, Paul G. Ince, Stephen B. Wharton, Dennis Wang, on behalf of the Cognitive Function and Ageing Neuropathology Study Group

**Affiliations:** 1 Sheffield Institute for Translational Neuroscience, University of Sheffield, Sheffield, S10 2HQ UK; 2 Department of Computer Science, University of Sheffield, Sheffield, S1 4DP UK; 3 Cambridge Public Health, Cambridge, CB2 1PZ UK; 4 Population Health Sciences Institute, Newcastle University, Newcastle upon Tyne, NE4 5PL UK; 5 Department of Psychiatry, University of Cambridge, Cambridge, CB2 0SP UK; 6 Singapore Institute for Clinical Sciences, A*STAR, Singapore, 117609 Singapore; 7 National Heart and Lung Institute, Imperial College London, London, SW3 6LY UK

**Keywords:** Dementia, Alzheimer’s, Feature selection, Machine learning, Neuropathology, Beta-amyloid

## Abstract

Although a variety of brain lesions may contribute to the pathological assessment of dementia, the relationship of these lesions to dementia, how they interact and how to quantify them remains uncertain. Systematically assessing neuropathological measures by their degree of association with dementia may lead to better diagnostic systems and treatment targets. This study aims to apply machine learning approaches to feature selection in order to identify critical features of Alzheimer-related pathologies associated with dementia. We applied machine learning techniques for feature ranking and classification to objectively compare neuropathological features and their relationship to dementia status during life using a cohort (*n*=186) from the Cognitive Function and Ageing Study (CFAS). We first tested Alzheimer’s Disease and tau markers and then other neuropathologies associated with dementia. Seven feature ranking methods using different information criteria consistently ranked 22 out of the 34 neuropathology features for importance to dementia classification. Although highly correlated, Braak neurofibrillary tangle stage, beta-amyloid and cerebral amyloid angiopathy features were ranked the highest. The best-performing dementia classifier using the top eight neuropathological features achieved 79% sensitivity, 69% specificity and 75% precision. However, when assessing all seven classifiers and the 22 ranked features, a substantial proportion (40.4%) of dementia cases was consistently misclassified. These results highlight the benefits of using machine learning to identify critical indices of plaque, tangle and cerebral amyloid angiopathy burdens that may be useful for classifying dementia.

**Supplementary Information:**

The online version contains supplementary material available at 10.1186/s13195-023-01195-9.

## Introduction

Dementia is a significant healthcare concern among the elderly, and the number of people with dementia will reach 131.5 million worldwide by 2050 [1]. There is no cure for this syndrome, but an accurate and timely diagnosis of dementia may create opportunities for patients to access symptomatic and potentially disease-modifying therapies. As defined in the Diagnostic and Statistical Manual of Mental Disorders 5th edition, cognitive and daily activity decline defines the syndrome, often measured using cognitive and functional tests along with medical history reported by the patient or caregiver [2]. In clinical settings, further investigations are performed primarily on younger onset dementias focused on anatomical and, sometimes, functional changes measured by magnetic resonance imaging (MRI) and positron emission tomography (PET) scans, and increasingly cerebrospinal fluid (CSF) samples taken from a lumbar puncture are considered to be dementia subtype biomarkers. However, dementia, as it most often manifests in older people, is associated with multiple brain pathologies [3, 4]. Research remains challenging when assessing the interactions among multiple brain factors related to the syndrome as it manifests during life.

The Cognitive Function and Ageing Studies (MRC CFAS, CFAS I, CFAS II) were longitudinal population-based ageing studies focusing on cognition. This analysis focused on brains donated from the original MRC CFAS. More than 550 participants from CFAS voluntarily donated their brains to the study after their death in order to undergo a comprehensive pathological assessment [5, 6]. Neuropathological investigations have explored the relationship of pathological features in the brain to dementia phenotypes, including various measures related to tau and beta-amyloid (Aβ) pathologies [7]. These studies showed considerable overlap in the burden of lesions between participants dying with and without dementia [3, 4]. Attributable risk showed the importance of many other pathologies in the brain [8, 9].

Machine learning (ML) classification algorithms and feature selection techniques have enabled automated ways of classifying heart and skin diseases and identified the most informative combination of predictors of those diseases [10, 11]. Studies investigating dementia involving brain imaging utilized three supervised ML algorithms (neural network, support vector machine and adaptive neuro-fuzzy inference system) for the diagnosis of Alzheimer’s disease (AD) and vascular dementia (VD) [12]. These algorithms used ranked MRI features based on their performance in identifying dementia cases within the dataset. Their results showed that categorizing AD and VD profiles using ML had high discriminant power with a classification accuracy of more than 84% in some cases. ML feature selection approaches were applied to enable the identification of neuropsychological measures and MRI features for the classification of AD [13]. ML using demographic and clinical features as predictors had also been used to predict dementia and neuropathology [14], but this assumes the predictors were stable over time. Alternatively, ML techniques could assess the relationship between dementia status and the neuropathological features of post-mortem brains and identify cases where they disagreed. Feature selection could also find which features are most informative of dementia. Where features are not informative, it could be interesting to reveal cases of dementia with insufficient pathology. Identifying informative features could help reduce resources, such as time, cost and effort utilized during pathological assessment and highlight a need for more profound clinical assessments.

In order to distinguish related indices such as plaque, tangle and CAA burdens, we needed an objective approach to rank these pathologies and identify a combination of features useful for classifying dementia. We hypothesized that ML feature ranking can identify a subset of neuropathological features ordered by their relative contribution to dementia. To test this hypothesis, we asked several questions during the analysis of neuropathological features: (1) How are they scored across dementia cases? (2) Are any features related to one another and convey redundant information? (3) Can we computationally rank the features in an unbiased way to facilitate ML? (4) What is the smallest subset of neuropathological features needed in an ML model to explain dementia? (5) Is there a limit to how accurately neuropathological features can classify dementia?

We investigated these questions using Alzheimer-related and other dementia-related pathologies measured in a population-representative subcohort of CFAS [6, 15–18]. There were 34 features determined by pathologists, including Aβ features, cerebral amyloid angiopathy (CAA) features and plaque scores. These features were automatically ranked, filtered and included in ML classifiers of dementia. We also reported the limits of ML classification of dementia using neuropathology factors and discussed possible reasons for these limitations.

## Material and methods

### Overview of the feature selection approach

**Fig. 1:**
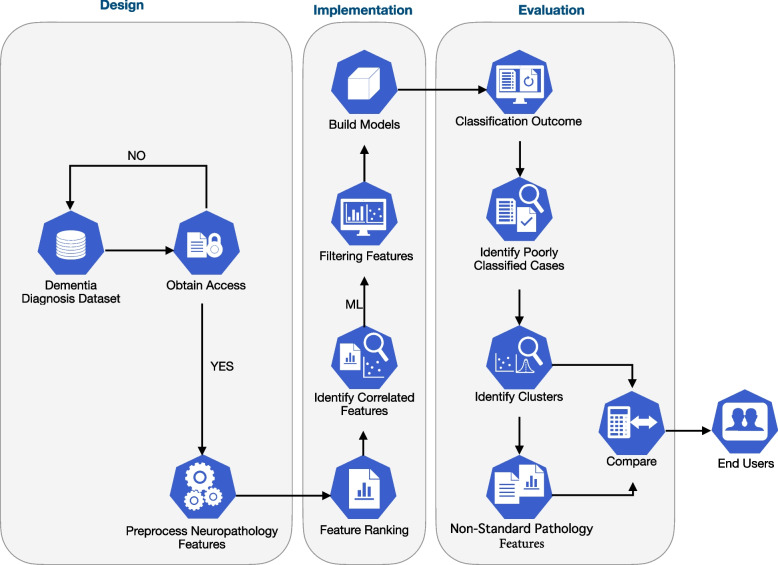
Methodology for classification of dementia. The methodology for the classification of dementia followed three stages: design, implementation and evaluation. First, we pre-processed and assessed feature-feature correlation after acquiring access to neuropathology and clinical data from CFAS. We then applied feature ranking methods to rank and filter all neuropathology features. Next, classifiers benchmarked with different subsets of features were selected according to their rankings. Finally, we compared cases that were consistently misclassified and evaluated brain attributes associated with these cases in order to improve machine learning

The selection of neuropathology features that were informative of dementia involved several steps (Fig. 1). We first obtained access to and downloaded the CFAS dataset following review and ethics approval by the CFAS management committee. Accordingly, a re-coding of available neuropathological features was performed to categorize and label them into distinct categories (tau, Aβ, demographics, etc.). We then applied supervised learning and feature selection techniques based on multiple filter-based methods. Features were ranked based on their importance and the most informative features were determined. The smallest subset of features that can classify dementia most accurately was identified using several ML classifiers. Finally, we examined misclassified cases in relation to the neuropathology features and linked the associations with other non-standard pathologies.

### Neuropathology features in the CFAS cohort

The CFAS cohort used for this study included data from two centres (Cambridge and Newcastle), totalling 186 subjects with 34 neuropathology features, plus age and brain weight, as shown in Table 1. Immunohistochemical detection of Aβ in formalin-fixed, paraffin-embedded sections (5 μm) is previously described [24]. Assessment of the Aβ phase was performed according to the Thal scheme and BrainNet Europe approach [21, 22]. Neurofibrillary tangles were assessed by the Braak stage [19] and plaques were assessed using the CERAD method [32]. The features included basic neuropathological measures for each subject, including Braak neurofibrillary tangle (NFT) stage, BrainNet Europe protocol for tau pathology, hippocampal tau NFT stage [26], Thal phase, primary age-related tauopathy (PART), cerebral amyloid angiopathy (CAA), thorn-shaped astrocytes (TSA) [17] and microinfarct stage [31] (Table 1).

**Table 1:** Description of the neuropathology features of CFAS in addition to the age and brain weight features

No.	Feature	Feature description	Type	Control		
				Dementia (***n***=107)	No dementia (***n***=70)	Missing (***n***=9)
**1**	Braak NFT stage	Braak stage refers to the Braak neurofibrillary tangle (NFT) stage (0–VI) [19, 20]	Nominal	107	70	0
**2**	Thal phase	Thal phase refers to the Thal Aβ phase, which is the new BrainNet stage for Aβ to detect immunopositive amyloid in cortical and subcortical areas and differentiate five phases [21, 22]	Nominal	107	70	0
**3**	Aβ stage typical	Aβ stage typical indicates the Aβ stage typical and atypical [18]	Nominal	107	70	0
**4**	PART-definite	PART relates to the new primary age-related-tauopathy concept. PARTdefinite as cases having no Aβ pathology (Thal 0) and with Braak NFT stages I–IV [23]	Nominal	50	47	80 (45.2%)
**5**	PART-all	Those cases with mild Aβ pathology (Thal I–II) and with Braak NFT stages I–IV [23]	Nominal	71	63	43 (24.3%)
**6**	CAA areas	The number of brain areas examined that have CAA (number of areas out of 9 maximum) [24]	Numeric	107	70	0
**7**	CAA type	As defined by Thal where CAA type 1 are cases with capillary amyloid and 2 only in larger vessels and type 0 no CAA [15, 24]	Nominal	107	70	0
**8**	CAA parenchymal	CAA severity score according to Love et al. [25] leptomeningeal and parenchymal vascular amyloid in four neocortical areas. So in any area, CAA can be 1, 2 or 3 and the score ranges from 0 to 12 [18]	Nominal	107	70	0
**9**	CAA meningeal	CAA severity meningeal has the same scoring system as CAA parenchymal with the score ranging from 0 to 12 [18]	Nominal	107	70	0
**10**	CAA total severity	The scores for parenchymal and leptomeningeal amyloid were summed in four areas, and scores range from 0 (minimum) to 24 (maximum) for severity in cortical areas [24]	Numeric	107	70	0
**11**	CAA frontal	CAA in the frontal cortex (present or absent) [26]	Nominal	107	70	0
**12**	CAA temporal	CAA in the temporal cortex (present or absent) [26]	Nominal	107	70	0
**13**	CAA parietal	CAA in the parietal cortex (present or absent) [26]	Nominal	107	70	0
**14**	CAA occipital	CAA in the occipital cortex (present or absent) [26]	Nominal	107	70	0
**15**	CAA hippocampus	CAA in the hippocampus and occipitotemporal gyrus (present or absent) [26]	Nominal	107	70	0
**16**	CAA cerebellum	CAA in the cerebellum (present or absent) [26]	Nominal	106	69	2 (1.13%)
**17**	BrainNet tau stage	BrainNet tau stage refers to BrainNet Europe protocol for tau pathology, a six-stage scheme that uses neuropil threads and is proposed by the BrainNet Europe Consortium [22]	Nominal	107	69	1 (0.6%)
**18**	Hippocampal tau NFT stage	Hippocampal tau neurofibrillary tangles (NFT) stage [26]	Nominal	56	35	86 (48%)
**19**	Subpial TSA in the expanded cortex	The subpial thorn-shaped astrocytes (TSA) in the expanded cortex	Nominal	107	69	1 (0.6%)
**20**	Subpial TSA in the mesial temporal lobe	The subpial thorn-shaped astrocytes (TSA) in the mesial temporal lobe	Nominal	107	69	1 (0.6%)
**21**	Subpial TSA in the brainstem	The subpial thorn-shaped astrocytes (TSA) in the brainstem	Nominal	107	67	3 (1.7%)
**22**	TSA-any	Thorn-shaped astrocytes (TSA) in any brain area (present or absent).	Nominal	107	69	1 (0.6%)
**23**	TSA-total	The number of areas in the brain with thorn-shaped astrocytes (TSA) [27–30]	Numeric	107	69	1 (0.6%)
**24**	Tufted astrocytes	The tufted parenchymal astrocytes in any brain area	Nominal	107	69	1 (0.6%)
**25**	Subpial mesial temporal	The subpial tau neurites in the mesial temporal lobe	Nominal	107	69	1 (0.6%)
**26**	Subpial brainstem	The subpial tau neurites in the brainstem/subcortical region	Nominal	107	67	3 (1.7%)
**27**	Argyrophilic grains	The argyrophilic grains disease	Nominal	107	69	1 (0.6%)
**28**	Cortical stage	The cortical microinfarcts stage which distinguishes the number of cortical areas that have microinfarcts	Numeric	106	70	1 (0.6%)
**29**	Subcortical stage	Subcortical lacune stage which distinguishes the number of subcortical areas that have microinfarcts	Numeric	106	70	1 (0.6%)
**30**	Microinfarct stage	The total microinfarct stage which differentiates the number of total areas that have microinfarcts	Numeric	106	70	1 (0.6%)
**31**	Frontal microinfarct	Frontal microinfarct [31]	Nominal	106	70	1 (0.6%)
**32**	Temporal microinfarct	Temporal microinfarct [31]	Nominal	106	70	1 (0.6%)
**33**	Parietal microinfarct	Parietal microinfarct [31]	Nominal	106	70	1 (0.6%)
**34**	Occipital microinfarct	Occipital microinfarct [31]	Nominal	106	70	1 (0.6%)
**35**	Age	Patient’s age at death	Numeric	107	70	0
**36**	Brain weight	Patient’s brain weight	Numeric	91	59	27 (15%)
**37**	Gender	Sex	Nominal	107	70	0
**38**	Virchow-Robin space expansion	Virchow-Robin spaces (VRS) are cavities filled with cerebrospinal fluid surrounding small penetrating cerebral arterioles with extensions of the subarachnoid space	Nominal	106	70	1 (0.6%)
**39**	Lewy bodies in substantia nigra	The Lewy body is a distinguishing neuronal inclusion. This is always found in the substantia nigra and brain regions in Parkinson’s disease, which occurs wherever there is excessive loss of neurons	Nominal	105	68	4 (2.3%)
**40**	Neuronal loss in the hippocampus	Neuronal loss in the hippocampus	Nominal	106	70	1 (0.6%)
**41**	Neuronal loss in substantia nigra	Neuronal loss in substantia nigra	Nominal	105	68	4 (2.3%)
**42**	Tangles in the temporal lobe	Tangles in the temporal lobe	Nominal	106	70	1 (0.6%)
**43**	Parenchymal CAA in the frontal lobe	Parenchymal CAA in the frontal lobe	Nominal	106	70	1 (0.6%)
**44**	Gliosis in the hippocampus	Gliosis in the hippocampus	Nominal	106	70	1 (0.6%)
**45**	Dementia status	Class label (dementia or no dementia) status of a patient	Binary	107	70	0

### Dementia status

Dementia status at death for each respondent was determined based on interviews/assessments during the last years of the respondent’s life. This included using the full Geriatric Mental State-Automated Geriatric Examination for Computer Assisted Taxonomy diagnostic algorithm, the Diagnostic and Statistical Manual of Mental Disorders (third edition-revised), interviews with the informants after the respondent’s death and the cause of death. Respondents were assessed as having no dementia at death if they had not been identified with dementia at their last interview less than 6 months before death or if they did not have dementia identified at the last interview and the retrospective interview showed no dementia at death. Bayesian analysis was used to estimate the probability of dementia when the last interviews were more than 6 months before death, and no record of having dementia at the interview and no retrospective informant interview (RINI) [5, 33]. A total of 107 of the 186 subjects had a diagnosis of dementia, which represented approximately 58% of the cohort. Of these 107 cases, 72 were women and 35 were men; their median ages were 89 and 88, respectively. There was a balanced gender ratio (37 females and 33 males) for participants dying without dementia (median age 85 and 79, respectively). The Consortium to Establish a Registry for Alzheimer’s disease (CERAD) criterion determined that in 64 out of the 107 cases (60.0%), Alzheimer’s disease was the definite, probable or possible cause of the observed symptoms.

### Ranking neuropathology features

We used several filter-based feature selection methods to determine the relevance of each feature to dementia in order to gain preliminary insight. These included chi-square (CHI) [34], gain ratio [35], information gain (IG) [36], reliefF [37, 38], symmetrical uncertainty [39], least loss [40] and variable analysis [41, 42]. Generally, filter-based methods use different mathematical models to compute feature relevance. These methods are efficient feature selection tools that employ mathematical models to derive scores for each feature based on correlations between the features and class labels in the input dataset. There can be discrepancies in the ranking of features based on such scores due to the different mathematical models used [42, 43]. The CFAS cohort consisting of 186 post-mortem and 34 neuropathology features was used for feature ranking. In addition to the 34 neuropathology features, age and brain weight were included. Using SciPy.stats v1.5.4 in Python3, we used *z*-score to adjust brain weight based on sex.

CHI utilizes the difference between observed and expected frequencies of the instances, as shown in Eq. (1).

1 $${X}^2=\frac{{\left(O-E\right)}^2}{E}$$

*O* and *E* are the observed and expected frequencies for a specific feature, respectively. IG employs Shannon entropy to measure the correlation between a feature and dementia status (Eqs. 2 and 3).

2 $$\textrm{IG}\ \left(S,A\right)=\textrm{Entropy}\ (S)-\sum \left(\left(\ |\ {S}_v\ |\div |\ S\ |\right)\times \textrm{Entropy}\ \left({S}_v\right)\right)$$

3 $$\textrm{where}\ \textrm{Entropy}\ (T)=-\sum {P}_c{P}_c$$

*P* is the probability that *S* belongs to class label *c*. *S*_*v*_ is the subset of *S* for which *a* feature has value *v.* |*S*_*v*_| is the number of data instances in *S*_*v*_, and |*S*| is the size of *S*.

A gain ratio is a normalized form of IG, which is estimated by dividing the IG by the Entropy of the feature with respect to the class (Eqs. 4 and 5).

4 $$\textrm{Gain}\ \textrm{ratio}=\frac{IG}{ENT\left(S,F\right)}$$

5 $$ENT\left(S,F\right)E=-\sum \frac{S_i}{S}{\mathit{\log}}_2\frac{S_i}{S}$$

where IG denotes the information gain, and *ENT* is the entropy of feature *F* over a set of examples *S*.

Symmetrical uncertainty deals with the bias of IG that occurs due to a large number of distinct values for the feature and presents a normalized score (Eq. 6).

6 $$SU\left(A,B\right)=\frac{2\times IG\left(A|B\right)}{E(A)+E(B)}$$

where *IG*(*A*| *B*) denotes the information gained by *A* after knowing the class. *E*(*A*) and *E*(*B*) are the entropy values of *A* and *B*, respectively.

ReliefF calculates the scores of each available feature with the class using the differences between the neighboring data instances and the target instances (Eq. 7).

7 $$W\left[A\right]=W\left[A\right]-\frac{\left( diff\frac{A,{R}_i,H}{m}\right)}{\ \left( diff\frac{A,{R}_i,M}{m}\right)}$$

where *W*[*A*] is the feature weights, *A* is the number of features, and *m* is the number of random training data instances out of the “*n*” number of training data instances used to amend *W*.

*R*_*i*_ = a randomly chosen test instance, and *H*/*M* is the nearest hit and nearest miss

Least loss is computed per feature based on the simplified expected and observed frequencies of the features (Eq. 8), and variable analysis employs a vector of scores of both CHI and IG results, normalizes the scores and then computes the vector magnitude (*V*_score) (see Eqs. 9 and 10).

8 $${L}^2\left(Y,X\right)={\sum}_{i,j}{\left[P\left({Y}_{i,}{X}_j\right)-P\left({Y}_i\right)P\left({X}_j\right)\right]}^2$$

where *X* is the independent feature class, *Y* is the class label, *P*(*Y*_*i*_) is the theoretical marginal distribution of 𝑌, and *P*(*X*_*j*_) is the theoretical marginal distribution of *X*, *P*(*Y*_*i*,_*X*_*j*_) is the theoretical joint probability distribution of *X* and *Y*.

9 $${V}_a=\left(\frac{IG_x}{CST_x}\right)$$

10 $$\left|{V}_a\right|=\sqrt{(IG)^2+{(TST)}^2}$$

where *V*_*a*_ is the square root of the sum of the square of its CHI and IG results of a feature.

The *V*_score and the correlation feature set results [44] are then integrated to represent a new measure of goodness to select relevant features.

2 $$IG\ \left(S,A\right)=\textrm{Entropy}\ (S)-\sum \left(\left(\ |\ {S}_v\ |\div |\ S\ |\right)\times \textrm{Entropy}\ \left({S}_v\right)\right)$$

The number of samples used in the feature selection process was 177 out of 186 after removing the nine missing values in the diagnostic class and 36 features (34 neuropathology features plus brain weight and age features). All filter-based feature selection was conducted using Waikato Environment for Knowledge Analysis (WEKA version 3.9.1) [45]. The percentage contribution of each feature was calculated by averaging the total weights assigned by all filter methods to each feature after normalizing weights scores.

### Dementia classification

We attempted the classification of dementia status in 146 samples after removing missing values from the 177 that were used in the feature selection process. The 146 samples had a slight class imbalance, with 89 demented versus 57 non-demented patients. Before training our models, we randomly selected 57 patients from the demented group using the sample() function from the random module in Python3. Then, the rows were shuffled using sklearn.utils version 0.22.2.post1. As a result, 114 samples were utilized after balancing the class label. The 32 samples were held out for final assessment. The hippocampal tau stage feature, which had 50% missing values, was dropped during the training process. Age and brain weight were removed before training the models, ending up with 22 features and 114 samples for classification. The dataset was split into a training set of 70% (80 samples) and a testing set of 30% (34 samples).

Seven classification algorithms were trained to classify individuals’ dementia status from the 22 top-ranked features. Scikit-learn version 0.22.2.post1 was used to implement and train the ML classifiers, and then measure their classification performance. Logistic regression was implemented using the *sklearn.linear_model* package where penalty was set to 12, the regularization parameter *C* was set to 1, the maximum number of iterations taken for the solvers to converge was set to 2000, and other parameters were set to default values. A decision tree classifier was implemented using the *sklearn.tree* package. K-nearest neighbors classifier was implemented using the *sklearn.neighbors* with the number of neighbors set to 5, the function “uniform weights” used for prediction, the “Minkowski” distance metric utilized for the tree, and with other parameters were set to default values. The linear discriminant analysis classifier was implemented using the *sklearn.discriminant_analysis* package with singular value decomposition for solver hyperparameter and other parameters were set to default values. The Gaussian naïve Bayes classifier was implemented using *sklearn.naive_bayes.* The support vector machine with a radial basis function kernel (SVM-RBF) was implemented using *sklearn.svm* with the regularization parameter *C* set to 1, the kernel coefficient gamma = “scale” and other parameters were set to default values. The support vector machine with a linear kernel (SVM-LINEAR) was implemented using the *sklearn.svm* package with regularization parameter *C* set to 1, with a “linear” kernel, gamma coefficient “scale” and other parameters were set to default. The *sklearn.metrics* package was used to report classification performance. Training and performance evaluation were performed 500 times, from which the average performance measure was calculated as overall performance. Accuracy, balanced accuracy, F1-score, precision, sensitivity and specificity utilizing regression plots were measures used for performance. ML models and feature selection libraries were built using Python 3.7.3.

### Classification with multiple feature sets

We created subsets of neuropathological features from the 22 top-ranked features in a step-wise manner to identify the smallest subset that included features with at least 5% contribution towards the classifier model. We initially created a feature set that contained the single top-ranked feature *N(1)*, which was used to train the ML algorithms to classify dementia and calculate their classification performances. Then, the second top-ranked feature was added to the feature subset to generate a feature set with *N(1)+1* features. The ML classifiers were trained using the new feature subset, and the classification performances were calculated. This process was repeated in descending rank order until a feature set containing all ranked features was included in the feature set. This process resulted in 22 feature sets that ranged in size from 1 to 22 features, with the performance of each feature subset in classifying dementia calculated. The best subset of features was determined as a compromise between performance and size. The data was split into a 30% test set and a 70% training set for each feature set.

### Evaluation of classification performance

We formulated the prediction of dementia as a binary classification problem (dementia, control); therefore, evaluation metrics, such as accuracy, F1-score, balanced accuracy, precision, specificity and sensitivity, were used to measure the performance of the subsets of features. The following evaluation metrics were used:

- True positives (TP): number of dementia cases that were correctly classified.
- False positives (FP): number of healthy subjects incorrectly classified as dementia cases.
- True negatives (TN): number of healthy subjects correctly classified.
- False negatives (FN): number of dementia cases incorrectly classified as healthy subjects.
- Accuracy (%): the proportion of correct classifications among total classifications:

11 $$\textrm{Accuracy}=\frac{\textrm{TP}+\textrm{TN}}{n}$$

where *n* is the number of total classifications per test.

- Sensitivity (%): The proportion of correctly classified dementia cases.

12 $$\textrm{Sensitivity}=\frac{\textrm{TP}}{\textrm{TP}+\textrm{FN}}$$

- Specificity (%): The proportion of correctly classified healthy subjects.

13 $$\textrm{Specificity}=\frac{\textrm{TN}}{\textrm{TN}+\textrm{FP}}$$

- Precision: The proportion of subjects classified as dementia cases who have dementia.

14 $$\textrm{Precision}=\frac{\textrm{TP}}{\textrm{TP}+\textrm{FP}}$$

- F1-score (F-measure) (%): Harmonic mean of precision and sensitivity.

15 $$\textrm{F}1=2\times \frac{\textrm{Sensitivity}\times \textrm{Precision}}{\textrm{Sensitivity}+\textrm{Precision}}=\frac{\textrm{TP}}{\textrm{TP}+\left(\textrm{FP}+\textrm{FN}\right)/2}$$

### Identifying misclassified cases

Leave-one-out cross-validation was used for training and performance evaluation of trained classifiers using Scikit-learn version 0.22.2.post1 [46] in Python3. A *split()* function was used to enumerate training and test sets for evaluation. The classification algorithms trained the classical AD features using the top-ranked 22 subsets and 114 samples, where one feature was added at a time creating 22 subsets of features for each classifier. All samples were clustered into true positive and true negative, false positive and false negative based on the performance of each classification run, and visualized using a heatmap to highlight the differences. The “*clustermap*” function in Seaborn package version 0.11.0 [46] was used for hierarchical clustering. The linkage method was set to average, and the distance metric was Euclidean.

### Explaining misclassified cases

To identify pathological and demographic features distinguishing the three clusters of classification performance, we used robust feature selection based on recursive feature elimination (RFE) with a linear SVM as the estimator [47] to identify the smallest set of non-standard pathological features for each of the three clusters [48]. This technique balances performance and computational cost [49]. The linear SVM was initially trained using the complete feature set of the training data with the *C*-parameter set to 1. The absolute weights in the weights vector of the hyperplane of the trained model were used to rank features according to importance, and the worst-performing feature was pruned from the feature set. This process was repeated until the required number of features in the signature was achieved. For a dataset with *J* samples and *K* features, *M*=100 subsamples were randomly sampled, feature selection was carried out in each subsample, and classification performance was calculated. For each cluster, different sizes of signatures ranged from one to the complete feature set. Each feature set was used to train an XGBoost model to classify the cluster against the rest [50]. The best signature of features for each cluster was chosen as a trade-off between signature size and classification performance. Accuracy and F1-score were used as classification metrics. ML models and feature selection libraries were built using Python 3.8.5, Scikit-learn 24.2 and Jupyterlab 2.2.6. We used the 114 samples and a “leave-one-out” cross-validation for training and performance evaluation of trained classifiers.

### Code availability

Links for python script codes in GitHub (https://github.com/mdrajab/CFAS-ranking-code) for the processes of ranking neuropathology features and classification models and (https://github.com/emmanueljammeh/cfas) for feature signatures showing association of the non-standard pathologies and demographics features with clusters.

## Results

### Distribution of neuropathology feature scores across dementia cases

**Fig. 2:**
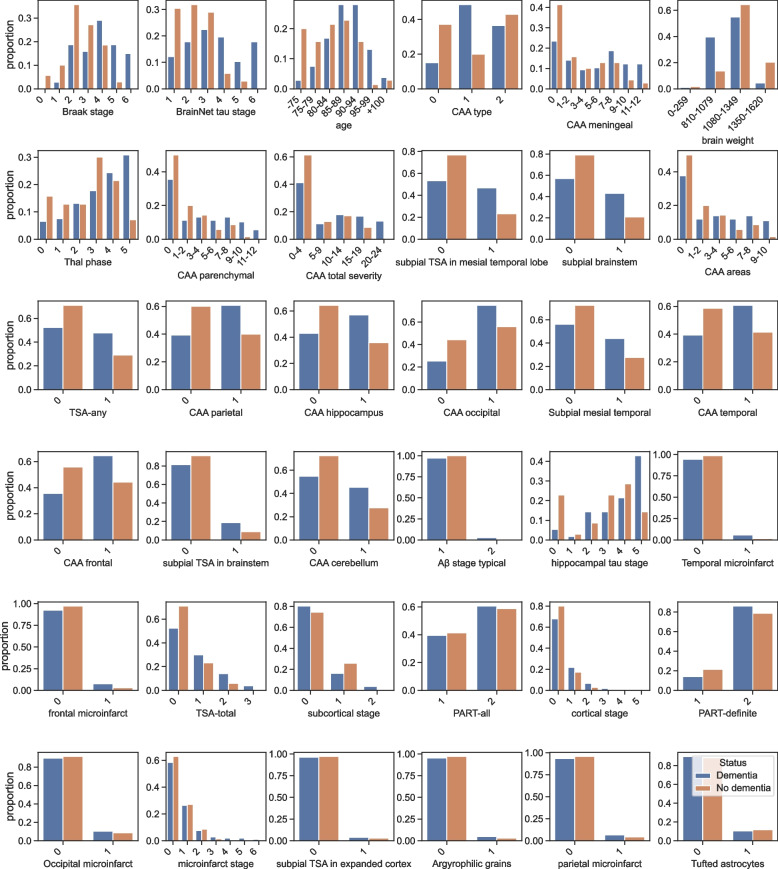
CFAS neuropathology feature distribution. The figure depicts neuropathology features distribution including age and brain weight (proportion of individuals with and without dementia of the CFAS neuropathology Dataset). All features shown were based on the ranking features list, from left to right. Most features were categorical, but some were ordinal, such as age, CAA total severity, brain weight, CAA areas, TSA-total, cortical stage, subcortical stage and microinfarct stage

Figure 2 depicts the distribution of values of participants dying with and without dementia across all neuropathological features in our study containing 186 samples and 34 attributes. In addition to the 34 neuropathological features, age and brain weight were included. People between 80 and 89 years had a higher frequency of dementia than other age sub-groups. The proportion of individuals with dementia increased with increasing Braak NFT stage, Thal phase and hippocampal tau stage. This validates previous findings from multivariable regression models of dementia and neuropathology [24]. The measures of CAA across subjects revealed that the proportion of dementia cases increased as the number of brain areas with CAA increased. Microinfarct features, in the frontal, occipital and parietal regions, were observed in individuals who died with dementia. A similar observation was seen with Aβ stage typical and Argyrophilic grains, which may limit classifiers from differentiating subjects using these features.

### Highly correlated neuropathology features

**Fig. 3:**
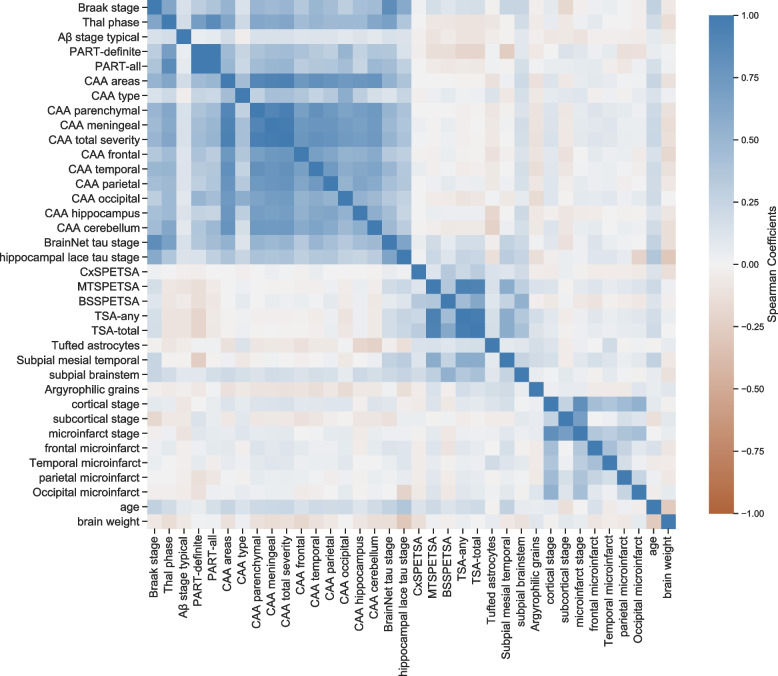
Spearman correlation of the complete CFAS neuropathological data set. Heat map of Spearman correlation coefficients between 34 neuropathology features in addition to age and brain weight features as a benchmark, 36 features in total and 186 samples. A coefficient close to 1 (blue colour) means a high positive correlation between the two variables. The diagonal line is the same variable, i.e. Spearman rho 1

The comparison of features identified highly correlated features (Spearman rho > 0.7), such as CAA-related features. Since CAA-related features, including CAA type, CAA areas and CAA total severity (CAA meningeal, CAA parenchymal), were shared among the top features presented by the different feature selection methods (Supplementary Table 1), we needed to ensure that only distinct features were chosen by minimizing feature-to-feature correlations. We identified three main clusters of highly correlated features (Fig. 3) when comparing all neuropathology features in our study. Hence, some of these features may be redundant for assessing dementia based on neuropathological features.

### Ranking of neuropathology features

The ranking of neuropathology features was conducted to estimate each feature’s contribution to dementia using seven feature ranking methods (Supplementary Table 1). A high ranking of the Braak NFT stage, which showed the neurofibrillary tangle stage (0–VI), supported it as a highly relevant feature for dementia pathology [19]. All ranking techniques (CHI, gain ratio, information gain, reliefF, symmetrical uncertainty, least loss and variable analysis) ranked the Braak NFT score in the top six, making it useful for human and computer-aided dementia diagnosis, and should be considered a primary attribute. Different feature selection techniques reported different rankings of the features; however, the most commonly used features were consistently highly ranked. For example, Braak stage, BrainNet tau stage, CAA type, Thal phase, subpial brainstem and subpial TSA in the mesial temporal lobe were consistently ranked in the top 12 (out of 36) notwithstanding which ranking method was used.

BrainNet tau stage appeared as the top of ranked features, and it had been previously found to be highly correlated with the Braak NFT stage as tangles and neuropil threads seemed to progress together [17]. BrainNet tau stage, a six-stage scheme that uses neuropil threads and was proposed by the BrainNet Europe consortium [51], has been used to predict dementia in recent research studies. CAA-related features, including CAA type, CAA areas and CAA total severity, were common among the top features presented by the different feature selection methods (Supplementary Table 1). We believed this may be partly due to the high correlation among these CAA-related features (Fig. 3). Therefore, we evaluated these features to ensure that only dissimilar features were chosen by minimizing feature-to-feature correlations. Lastly, subpial TSA in the mesial temporal lobe appears frequently in the results of all feature selection methods with a high rank. This indicated that the presence of subpial TSA in the mesial temporal lobe had a strong association with dementia.

**Fig. 4:**
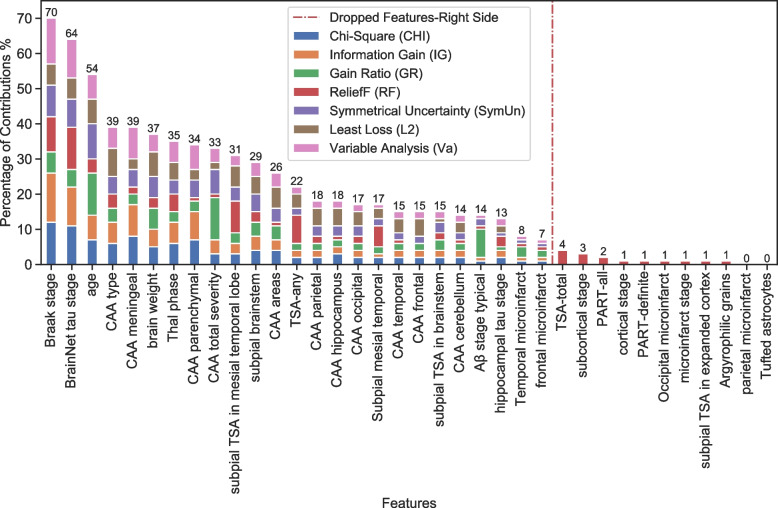
Ranking of neuropathology features. Ranking 34 neuropathology features plus age and brain weight using seven filter methods. After normalizing the weight scores of each feature, the percentage contribution of each feature was calculated by averaging the total weights assigned to each feature by all filter methods. The dotted line indicates features to be dropped, which features percentage contribution show less than 7%

All 34 neuropathology features, in addition to age and brain weight, and 186 samples were assessed using seven ranking methods (Supplementary Table 1; Fig. 4). We calculated each feature’s contribution percentage based on each ranker’s weights. We did this by taking each feature’s average of the total weight assigned by all filter methods. All features, except parietal microinfarct and Tufted astrocytes, were estimated by one ranking method to have at least 1% contribution to dementia classification. We found a subset of 25 features where all ranking methods estimated a percentage of contribution and at least 5% contribution. In order to assess the utility of neuropathology features to classify dementia, we removed the non-neuropathology features (age and brain weight) and hippocampal tau stage due to high missingness, leaving 22 top-ranked features.

### Classification of the ranked neuropathology features

We further investigated subsets of the top 22 ranked neuropathological features and 114 samples using ML classification. A single feature was successively added from the 22 top-ranked feature set to create subsets with sizes ranging from 1 to 22 (from top to lower-ranked features). The dataset was randomly split into a training set containing 70% of the samples and the remaining 30% was used for testing. The training set was used to train classification models using logistic regression, decision tree, k-nearest neighbors, linear discriminant analysis, Gaussian naïve Bayes, SVM-RBF and SVM-LINEAR classification algorithms. The performance of each trained model was evaluated using the test set for prediction. Supplementary Fig. 1 depicts the F1-score performance of all subsets of features (by forward and backward order of ranked features) in classifying dementia status for the seven ML classifiers considered. In the F1-score, the top eight features had the highest performance of 74% using the algorithms SVM-RBF and logistic regression. For comparison with a traditional univariate approach, we trained each neuropathology feature using the seven classifiers and reported their F1-scores. The Thal phase was found to have achieved a 69% F1-score using SVM-LINEAR (Supplementary Fig. 2). The results were supported by the accuracy and balanced accuracy that showed the top eight features’ achieving 74% with most classifiers (Supplementary Figs. 3 and 4). There was no significant improvement in classification beyond the use of eight features. As the number of features was increased beyond eight, most of the trained models performed slightly worse in identifying dementia patients, possibly due to overfitting. We also showed sensitivity and specificity for all models to explain why some of the forward-ranking performances increased when adding the last three features (Supplementary Figs. 5 and 6). Some of these had class imbalance, resulting in high specificity but low sensitivity. For example, in the linear discriminant analysis classifier, the last five features achieved 84% sensitivity but 50% specificity.

### Limits to the accuracy of classification of neuropathology features

Classification results of different feature subsets using the seven classifiers, 114 samples and 22 top-ranked neuropathology features showed that 40.4% of patients were misclassified out of 114 individuals using cross-validation. Furthermore, we investigated the cause of the high misclassification rate. Heatmaps used to visualize the classification of each patient revealed that some cases were misclassified as false positives or negatives, irrespective of the machine learning algorithm used. Supplementary Fig. 7 shows the clustering of patients classifications from seven classification techniques using multiple subsets of features in order to identify similarities in their performance. Three clusters were identified, containing cases classified correctly, and misclassified as a false positive or false negative. The false positive cluster denoted cases where neuropathology features classified them as having had dementia when in actuality, they did not. Conversely, the false negative cluster denotes cases classified as not having dementia, but in reality, they did. Perhaps, this cluster could correspond to cases of dementia with insufficient neuropathology changes [52].

For each misclassified case (false positive or false negative), we looked at the Mini-Mental State Exam (MMSE) scores at baseline and final interviews (Supplementary Fig. 8). For false negatives, there were observations of more moderate and severe cases at the final interview compared with baseline. On the other hand, the false positives were evenly distributed as normal, mild and moderate at baseline, with no severe cases. Then, we performed further analyses to determine which features were associated with cases where the ranked neuropathology features alone could not explain dementia. Since the classical markers of neuropathology features summarizing the prevalence of plaques and tangles did not classify a large proportion of patients, we hypothesized that non-standard pathologies for rarer dementia syndromes and regional markers could be more helpful. These less common and “disregarded” pathologies have been described across the CFAS cohort [53]. The non-standard features used were based on more granular neuropathology features in different regions in the brain, such as neuronal loss, gliosis, pick bodies, Lewy bodies, spongiform changes, superficial gliosis, tangles, Virchow-Robin space expansion and ballooned neurons and some demographic features such as gender, age and brain weight features.

**Fig. 5:**
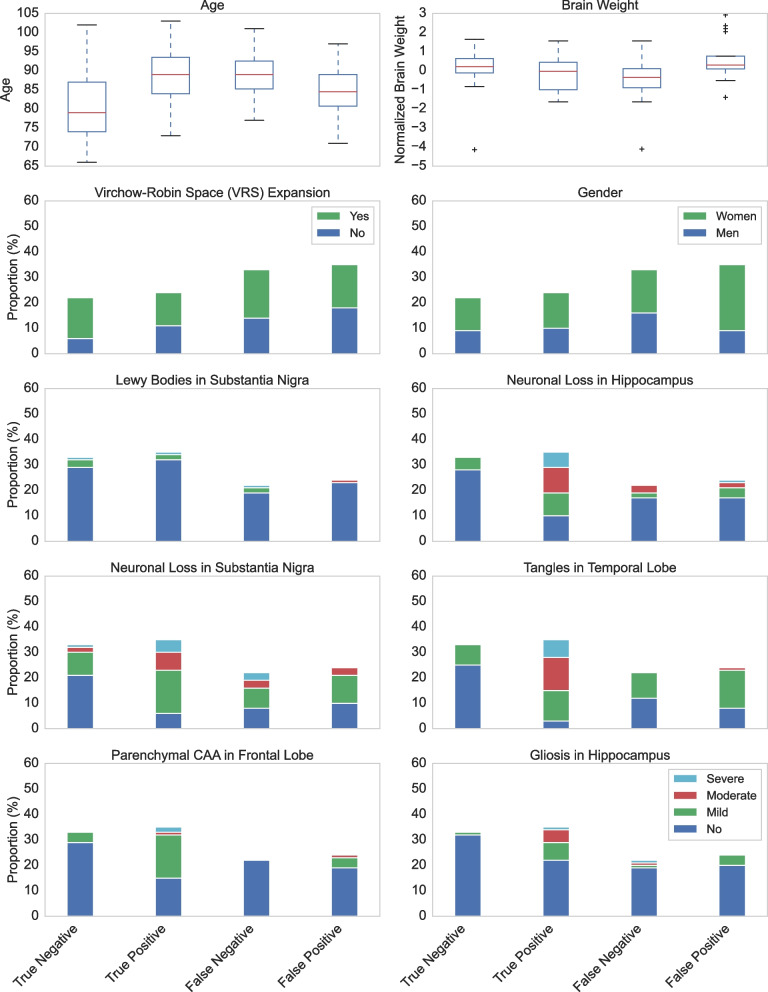
Classification performance of standard and non-standard neuropathological and demographic features. Non-standard neuropathological and demographic features were associated with misclassified and correctly classified cases by classifiers that used the standard neuropathology features

Our best-performing model for non-standard features, SVM-RFE, effectively removed irrelevant and redundant features to achieve good generalization. The level of each non-standard feature was compared to the classification performance of the classifiers using standard neuropathology (Fig. 5). We found that the mean age for false negative cases was the highest, with a mean of 89.3 years. In contrast, the false positive mean age was 84.5, and the true positive and true negative mean ages were 88.5 and 80.6, respectively. We also found that the mean brain weight was lower in the false negative cases than in the false positives, true positives and true negatives. Lewy bodies in the substantia nigra, neuronal loss in the hippocampus, neuronal loss in the substantia nigra, tangles in the temporal lobe, parenchymal CAA in the frontal lobe and gliosis in the hippocampus could all be combined to explain the classification performance of standard neuropathology (Supplementary Fig. 9). However, a high proportion of misclassifications occurred where there was a lack of any pathology (Supplementary Fig. 10). A *t*-test of each feature also demonstrated no difference in the values of non-standard pathology features between false positives and negatives (Supplementary Table 2).

For further evaluation, we combined the top eight classical neuropathological features with the ten non-standard features associated with classifier performance. Together, we tested subsets of the 18 features to classify dementia status. When using classical features, we observed that 40.4% of cases were misclassified; however, when the feature sets were combined, the misclassified cases decreased to 35.1% (Supplementary Fig. 10). The decrease in misclassification was observed in individuals of at least 85 years old (46.3 to 40.3%) and in those younger than 85 years (31.9 to 27.7%). Of the 32 cases held out, we observed a sensitivity of 68.8% (logistic regression) using the top eight neuropathology features. In contrast, the combined standard and non-standard neuropathological features achieved a better sensitivity of 81.3%.

## Discussions and conclusions

In this study, we introduced an ML approach to describe how neuropathological features at the end of life were related to dementia. Our step-wise ML approach to rank and select Alzheimer-related pathologies allowed us to investigate how the different measures, such as those related to Aβ-related assessments and tau, can inform about dementia status. The different feature ranking methods resulted in a slightly different ordering of the features in terms of their association with dementia status. However, the top-ranked features were consistent across methods. For example, the Braak NFT and BrainNet tau stages were the top two selected features in line with previous studies [6, 17, 18, 54, 55]. However, our results also showed that subpial TSA in the mesial temporal lobe was highly ranked, presenting a contradictory finding from prior studies [6]. Additionally, we identified three clusters of highly correlated measures in the dataset, CAA, TSA and microinfarct-related, demonstrating that some measures were redundant. Removing these redundant features may reduce collinearity and improve the performance of feature selection and classification accuracy [56–60].

In order to examine the impact of ranking, we tested seven classification algorithms using different subsets of ranked features. Cross-validation during classifier training yielded a maximum classification accuracy of, at most, 74%, using the top eight ranked features. Two sub-groups of misclassified participants were identified (false positives and negatives), accounting for 21.2% and 19.3%, respectively. These individuals were consistently misclassified across all classification algorithms. In order to improve classification accuracy, we also considered whether more specific neuropathology features for particular brain regions, which were collected in addition to the standard assessment, could help with classification. Consistent with previous reports, dementia was most associated with age and brain weights [4]. We further found that the classification of dementia using AD pathology differed between younger and older individuals [8]. Our results suggested that imaging and body fluid biomarkers for a range of pathological changes should be used to identify pathophysiologic processes associated with dementia in individual patients [61–64]. The feature ranking and filtering approaches could be applied to these other sources of pathology data.

The high proportion of misclassifications (35.1%) also indicated discordance between neuropathology and dementia, where some demented individuals had no known pathology and some non-demented individuals with pathology. An explanation for the poor classification performance is that some cases express dementia during life without classical neuropathological changes [52]. Corrada et al. reported that 22% of demented participants did not have sufficient pathology to account for cognitive loss [65]. Using the Vantaa 85+ cohort, Hall et al. showed that cognition and education predicted dementia but not AD or amyloid-related pathologies in the elderly [14]. When combining the top eight neuropathology features with the non-standard pathologies’ features, the discordance was less for older individuals (85 years old and above).

The results can be further investigated using other ML techniques, such as embedded feature selection and additional cohorts with the same pathology features and clinical outcomes. Alzheimer’s Disease Neuroimaging Initiative [66] or the Rush Memory and Ageing Project [67] could be cohorts to validate our findings from CFAS. However, this requires adjusting for demographic and measurement differences between these other cohorts. Another challenge in relating neuropathology assessments to the clinical diagnosis of dementia was the time lapse between the last assessment of dementia and the post-mortem assessment of the brain. Further follow-up reports on the participant’s cognitive status could be collected from those who knew the individual up to the time of death. Pathological features may differ between different types of dementia, such as AD, frontotemporal dementia, vascular disease and Lewy body dementia [68–70]. There is a need to quantify measures of other key age-related brain pathologies, particularly vascular disease, synuclein staging and age-related transactive response DNA-binding protein 43 (TDP43) pathology (limbic predominant age-related TDP43 encephalopathy). By doing so, we could link pathology with other symptoms related to dementia. Rather than assessing associations between one feature and an outcome at a time, it would be helpful to investigate whether combinations of features were associated with dementia [71–75].

This study provided a new approach to understanding how much cognitive classification of dementia can be explained by pathological features of the brain. The application of ML as a means of robust evaluation of neuropathological assessments and scores for 186 subjects and 34 neuropathology features from the CFAS cohort highlighted key indices of Alzheimer-related pathologies that may contribute to dementia. While we found that as many as 22 neuropathology features could be independently associated with dementia, tau-related assessments were most informative for ML classifiers of dementia. We hope that further neuropathology studies using multiple feature ranking techniques can lead to identifying more robust biomarkers and enhance the early detection of disease.

## Supplementary Information

**Additional file 1: Supplementary Figure 1.** F1-score performance of all subsets of neuropathology features. **Supplementary Figure 2.** F1-score performance of each single neuropathology feature from the rank list. **Supplementary Figure 3.** Accuracy performance of all subsets of neuropathology features from the rank list forward and backward rankings. **Supplementary Figure 4.** Balanced Accuracy performance of all subsets of neuropathology features from the rank list forward and backward rankings. **Supplementary Figure 5.** Sensitivity performance of all subsets of neuropathology features from the rank list forward and backward rankings. **Supplementary Figure 6.** Specificity performance of all subsets of neuropathology features from the rank list forward and backward rankings. Supplementary Figure 7. Clustering of classification performance. **Supplementary Figure 8.** Distribution of MMSE scores. **Supplementary Figure 9.** Non-standard neuropathological and demographic features. **Supplementary Figure 10.** Clustering of 18 features, including eight top-ranked neuropathology features and ten non-standard neuropathology features. **Supplementary Table 1.** Ranking of the CFAS Dataset Features. **Supplementary Table 2.** T-test and p-values for all non-standard and demographic features.

## Data Availability

Data from the CFAS study is accessible via application to the CFAS (http://www.cfas.ac.uk/cfas-i/data/#cfasi-data-request), under the custodianship of FM and CB.
