# Supplementary material for: Assessment of Alzheimer-related pathologies of dementia using machine learning feature selection
Source: Alzheimers Res Ther. 2023 Mar 10;15:47. doi: 10.1186/s13195-023-01195-9 (PMC9999590; doi:10.1186/s13195-023-01195-9)
Supplement: Supplementary file 1 — Additional file 1: Supplementary Figure 1. F1-score performance of all subsets of neuropathology features. Supplementary Figure 2. F1-score performance of each single neuropathology feature from the rank list. Supplementary Figure 3. Accuracy performance of all subsets of neuropathology features from the rank list forward and backward rankings. Supplementary Figure 4. Balanced Accuracy performance of all subsets of neuropathology features from the rank list forward and backward rankings. Supplementary Figure 5. Sensitivity performance of all subsets of neuropathology features from the rank list forward and backward rankings. Supplementary Figure 6. Specificity performance of all subsets of neuropathology features from the rank list forward and backward rankings. Supplementary Figure 7. Clustering of classification performance. Supplementary Figure 8. Distribution of MMSE scores. Supplementary Figure 9. Non-standard neuropathological and demographic features. Supplementary Figure 10. Clustering of 18 features, including eight top-ranked neuropathology features and ten non-standard neuropathology features. Supplementary Table 1. Ranking of the CFAS Dataset Features. Supplementary Table 2. T-test and p-values for all non-standard and demographic features. [file 13195_2023_1195_MOESM1_ESM.docx]

**Supplementary Materials**

**Supplementary Figure 1 F1-score performance Performance of all subsets of neuropathology features**
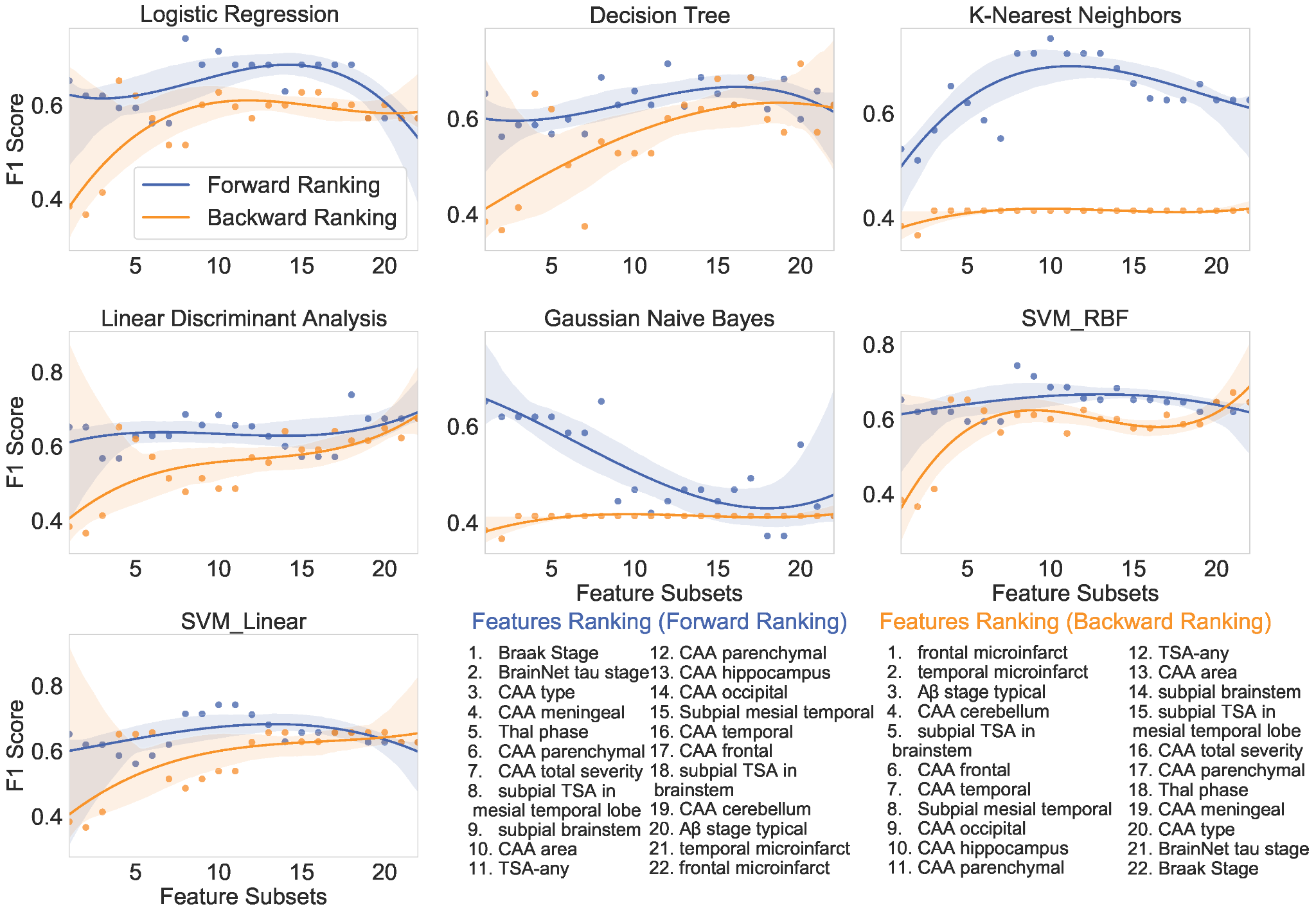
Supplementary Figure 1. Performance of all subsets of neuropathology features. F1-score performance of all subsets of neuropathology features from the rank list forward and backward rankings. Forward ranking (blue) adds to the classifier model from the top feature to the lowest feature, while backward ranking (orange) adds to the model from the lowest feature to the top feature. Seven classifiers were utilized in this investigation: logistic regression, decision tree, k-nearest neighbors, linear discriminant analysis, gaussian naive bayes, support vector machines with radial basis function kernel, and support vector machines with linear kernel. Please see (Supplementary: Figures 3-6) for other metrics such as accuracy, balanced accuracy, sensitivity, and specificity.

**Supplementary Figure 2 F1-score performance of each single neuropathology feature from the rank list**


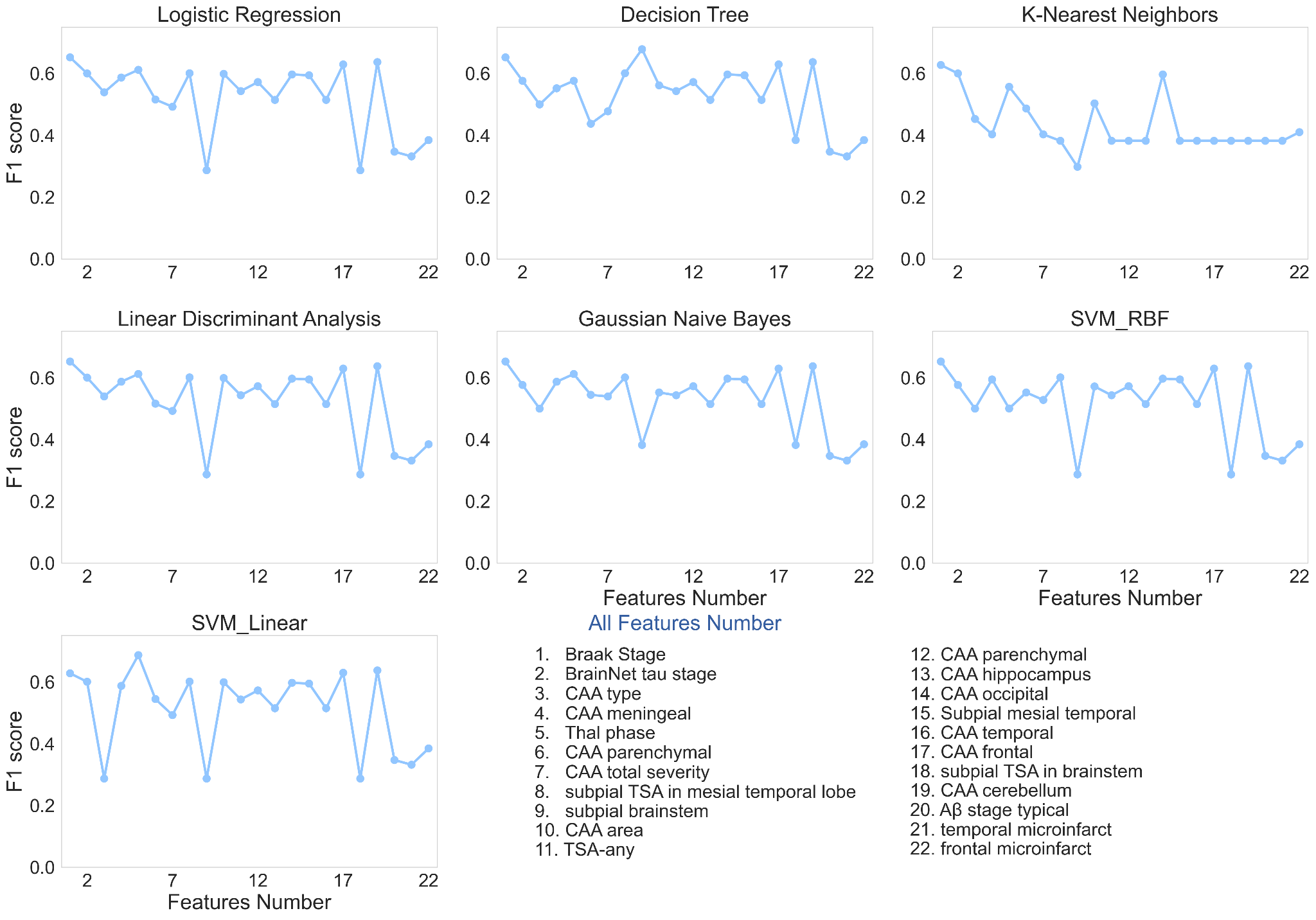
 Supplementary Figure 2. F1-score performance of each single neuropathology feature from the rank list. This is to show comparison of a traditional univariate approach. Seven classifiers were utilized in this investigation with top score in each: Logistic Regression (Braak stage: 0.65), Decision Tree (Subpial Brainstem: 0.68), k-Nearest Neighbors (Braak stage: 0.63), Linear Discriminant Analysis (Braak stage: 0.65), Gaussian Naive Bayes (Braak stage: 0.65), Support Vector Machines with Radial Basis Function kernel (Braak stage: 0.65), and Support Vector Machines with Linear kernel (Thal phase: 0.69).

**Supplementary Figure 3 Accuracy performance of all subsets of neuropathology features from the rank list forward and backward rankings**

*
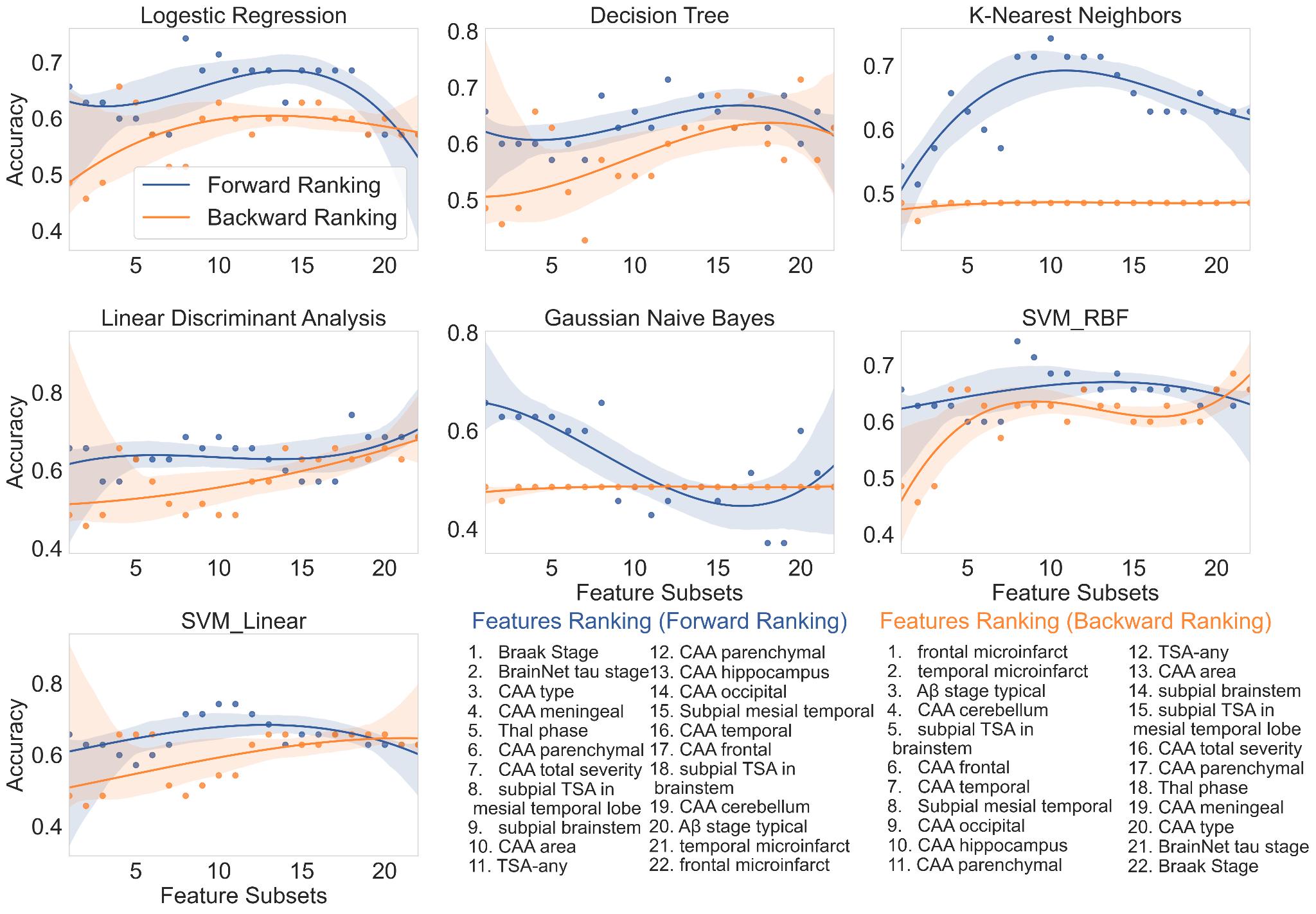
*

Supplementary Figure 3. Accuracy performance of all subsets of neuropathology features from the rank list forward and backward rankings. Forward ranking (blue) adds to the classifier model from the top feature to the lowest feature while the backward ranking (orange) adds to the model from the lowest feature to the top feature. Seven classifiers were utilized in this investigation: Logistic Regression, Decision Tree, k-Nearest Neighbors, Linear Discriminant Analysis, Gaussian Naive Bayes , Support Vector Machines with Radial Basis Function kernel, and Support Vector Machines with Linear kernel.

**Supplementary Figure 4 Balanced Accuracy performance of all subsets of neuropathology features from the rank list forward and backward rankings**

*
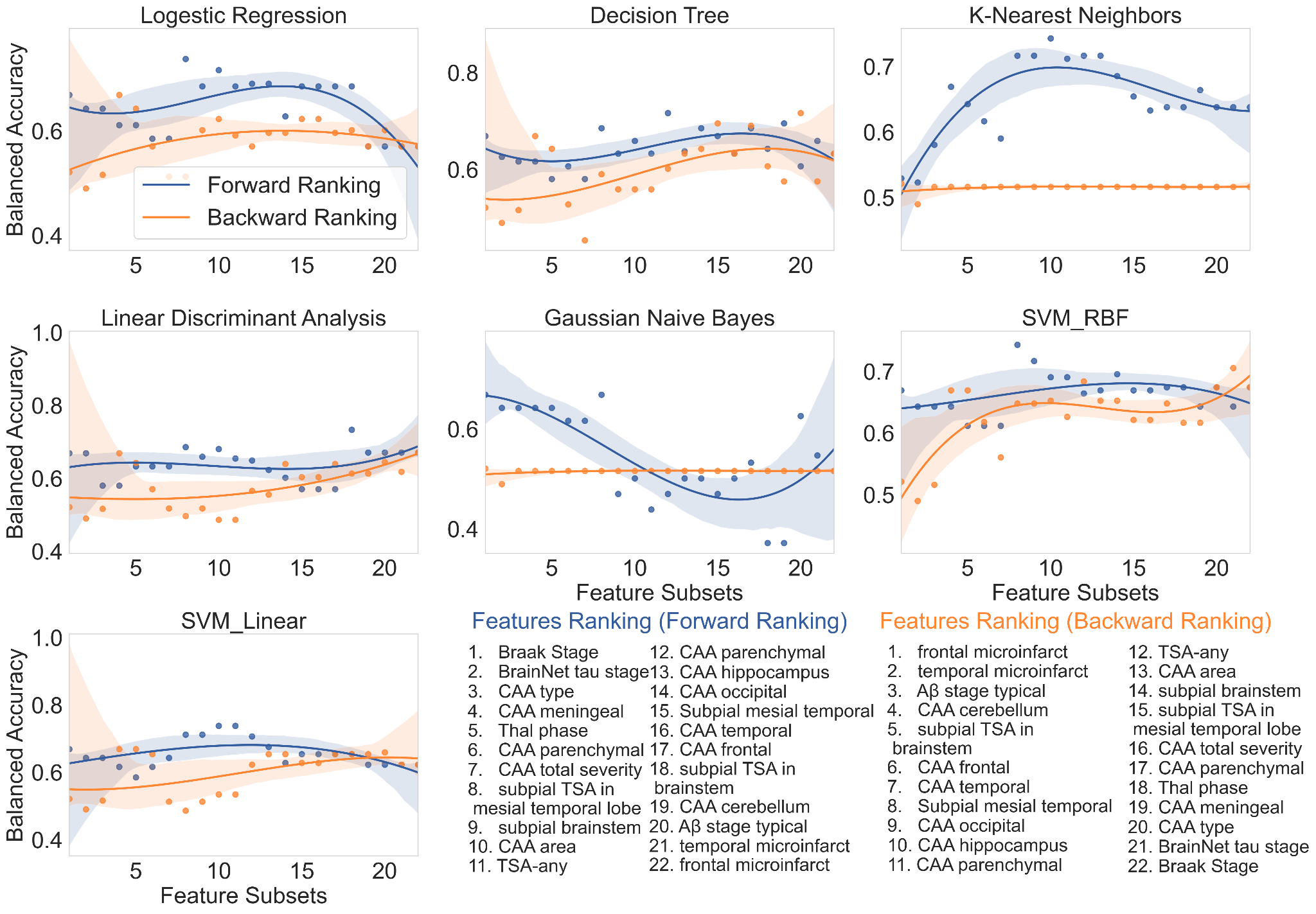
*

Supplementary Figure 4. Balanced Accuracy performance of all subsets of neuropathology features from the rank list forward and backward rankings. Forward ranking (blue) adds to the classifier model from the top feature to the lowest feature while the backward ranking (orange) adds to the model from the lowest feature to the top feature. Seven classifiers were utilized in this investigation: Logistic Regression, Decision Tree, k-Nearest Neighbors, Linear Discriminant Analysis, Gaussian Naive Bayes , Support Vector Machines with Radial Basis Function kernel, and Support Vector Machines with Linear kernel.

**Supplementary Figure 5 Sensitivity performance of all subsets of neuropathology features from the rank list forward and backward rankings**

*
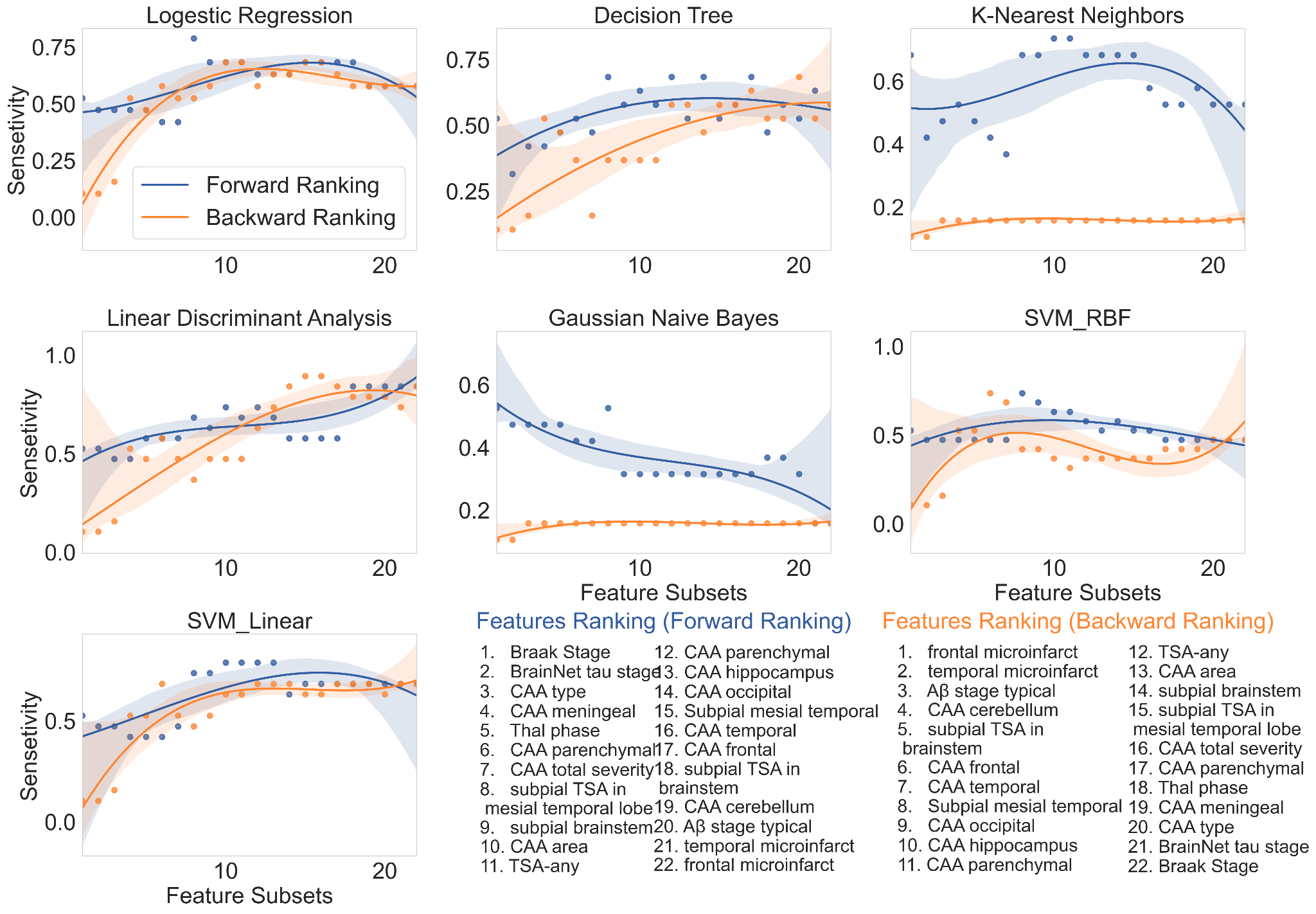
*Supplementary Figure 5. Sensitivity performance of all subsets of neuropathology features from the rank list forward and backward rankings. Forward ranking (blue) adds to the classifier model from the top feature to the lowest feature while the backward ranking (orange) adds to the model from the lowest feature to the top feature. Seven classifiers were utilized in this investigation: Logistic Regression, Decision Tree, k-Nearest Neighbors, Linear Discriminant Analysis, Gaussian Naive Bayes , Support Vector Machines with Radial Basis Function kernel, and Support Vector Machines with Linear kernel.

**Supplementary Figure 6 Specificity performance of all subsets of neuropathology features from the rank list forward and backward rankings**

*
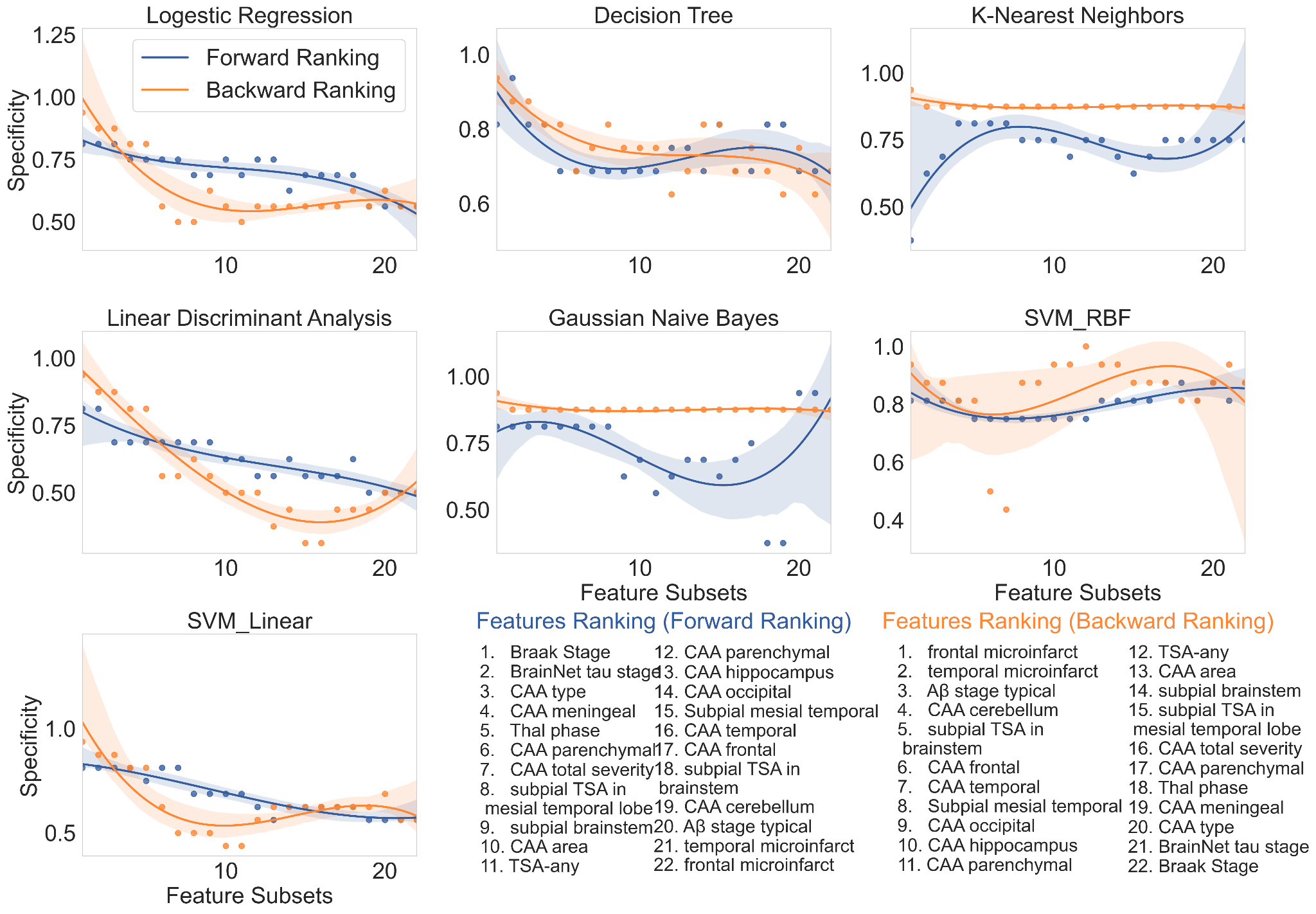
*Supplementary Figure 6. Specificity performance of all subsets of neuropathology features from the rank list forward and backward rankings. Forward ranking (blue) adds to the classifier model from the top feature to the lowest feature while the backward ranking (orange) adds to the model from the lowest feature to the top feature. Seven classifiers were utilized in this investigation: Logistic Regression, Decision Tree, k-Nearest Neighbors, Linear Discriminant Analysis, Gaussian Naive Bayes , Support Vector Machines with Radial Basis Function kernel, and Support Vector Machines with Linear kernel.

**Supplementary Figure 7 Clustering of classification performance**


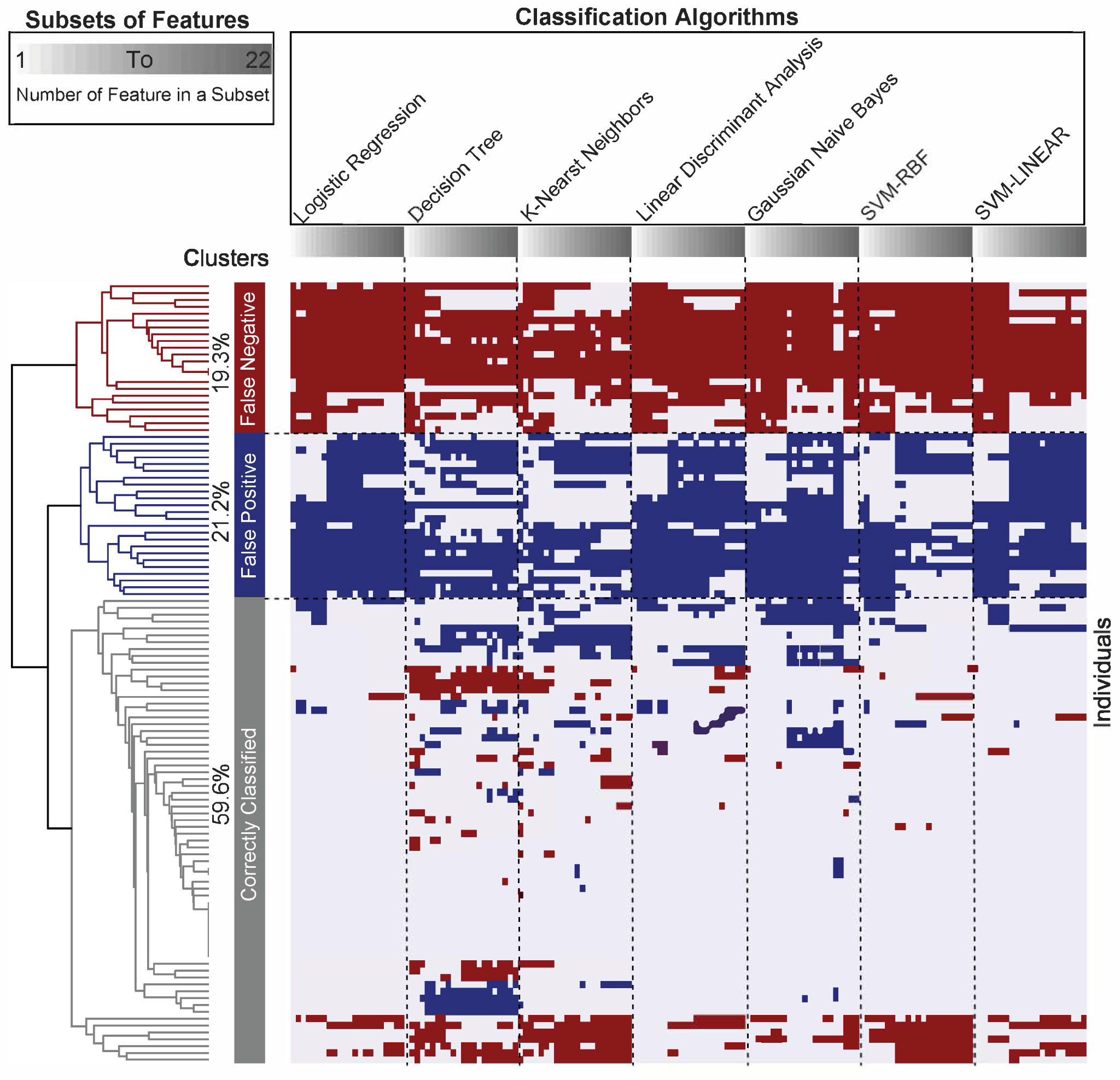


Supplementary Figure 7. Clustering of classification performance. Clustering of classification performance from leave one out cross-validation on 114 CFAS participants and top 22 ranked standard neuropathology features. Each cluster illustrates a classification that was given to individuals consistently or nearly consistently, irrespective of what classification algorithm was used. Evaluation of 7 classifiers revealed 24 individuals (blue) were mostly misclassified as a false positive, 22 individuals (red) were mostly misclassified as false negative, and 68 individuals (grey) were mostly correctly classified as true positive or true negative. Each algorithm evaluated subsets of ranked features from 1 (top feature) to 22 features (all ranked features).

**Supplementary Figure 8 Distribution of MMSE scores**


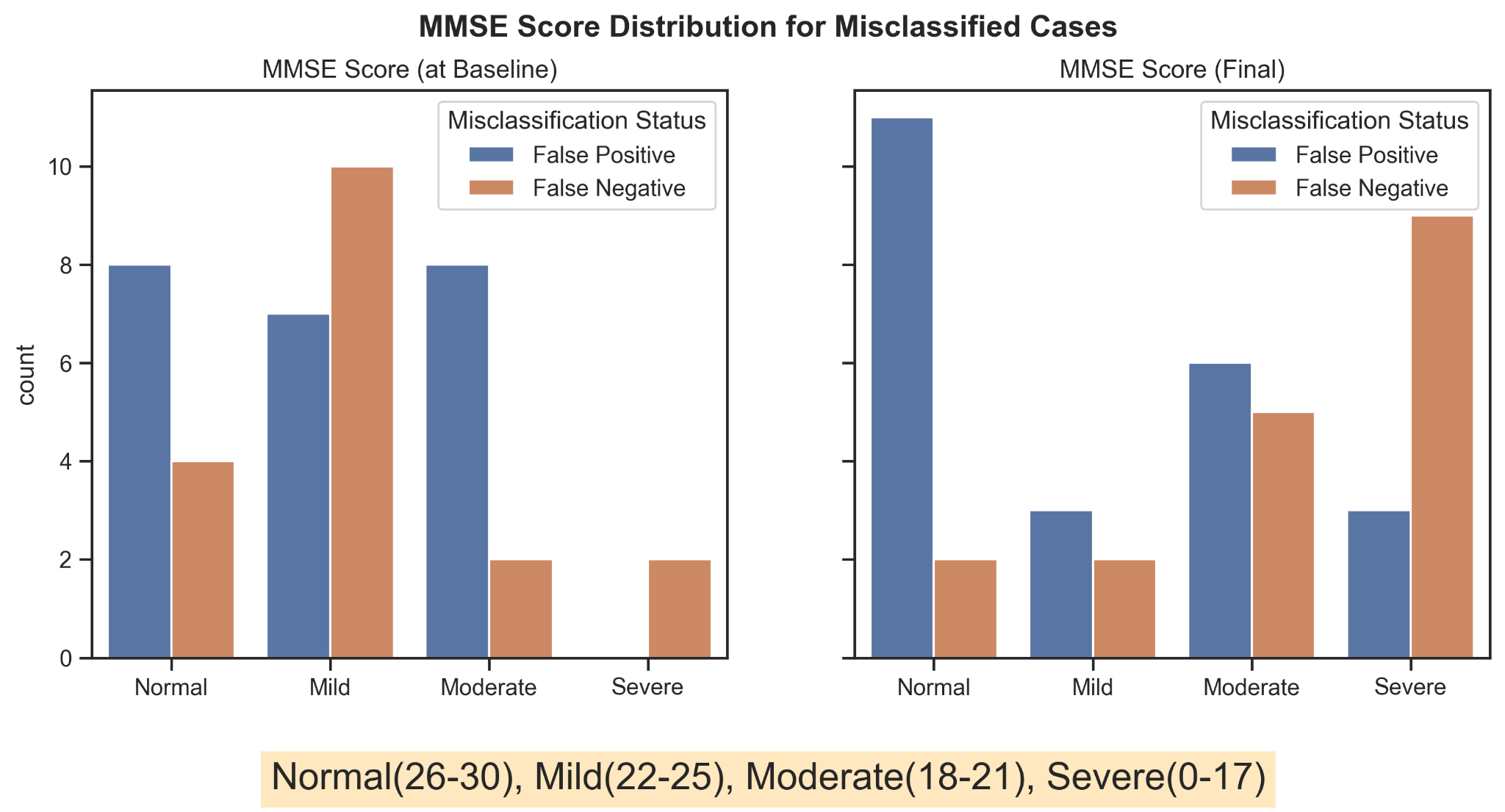


Supplementary Figure 8. Distribution of MMSE scores at baseline and final for all misclassified cases (False positive and False Negative).

**Supplementary Figure 9 Non-standard neuropathological and demographic features**


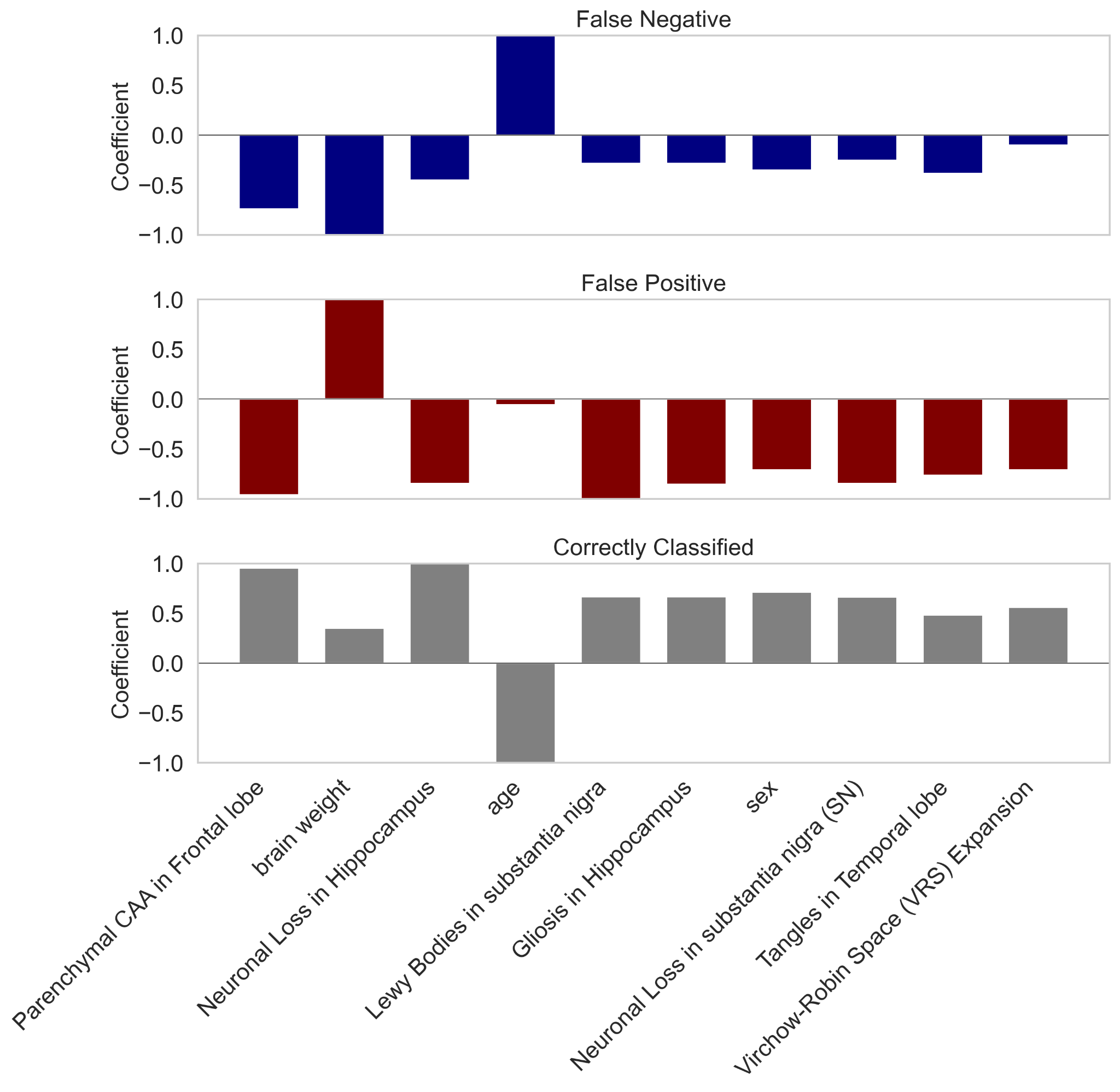
Supplementary Figure 9. Non-standard neuropathological and demographic features that were associated with misclassified and correctly classified cases by the classic neuropathology features. The coefficients shown for each variable were extracted from the most predictive support vector machine classifiers: demographics features such as age, brain weight and sex.

**Supplementary Figure 10 Clustering of 18 features, including eight top-ranked neuropathology features and ten non-standard neuropathology features**

####
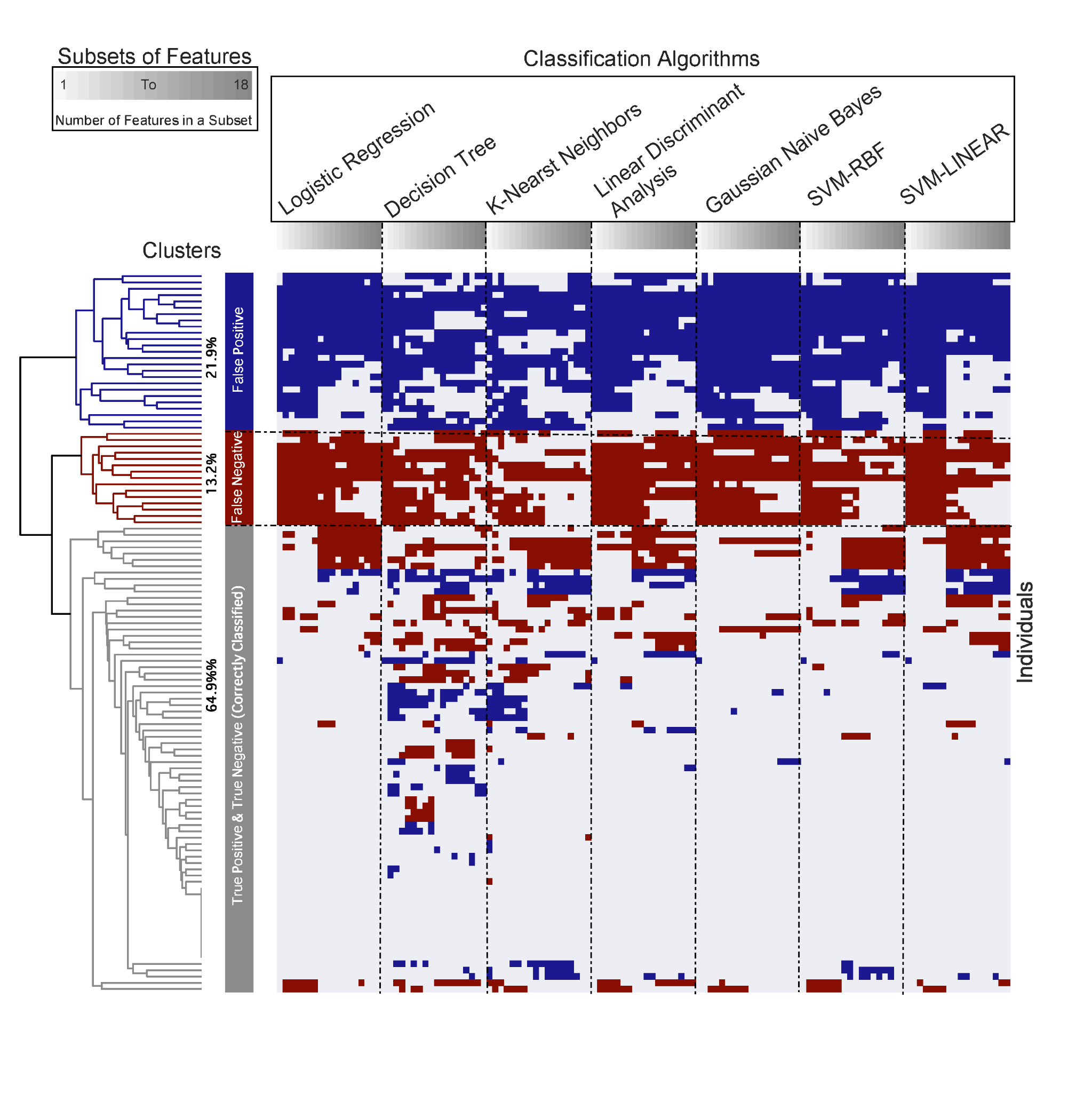


Supplementary Figure 10. Clustering of cross-validation classification performance on 114 CFAS participants and subsets of 18 features, including eight top-ranked neuropathology features and ten non-standard neuropathology features. Each cluster illustrates a classification that was given to individuals consistently, or nearly consistently, irrespective of what classification algorithm was used. Evaluation of 7 classifiers revealed 23 individuals (blue) were mostly misclassified as false positive, 15 individuals (red) were mostly misclassified as false negative, and 76 individuals (grey) were mostly correctly classified as true positive or true negative.

####

####

**Supplementary Table 1 Ranking of the CFAS Dataset Features**

| **NO** | **Chi-Squares** | **Gain Ratio** | **Information Gain** | **ReliefF** | **Symmetrical Uncertainty** | **Least Loss** | **Variable Analysis** |
| --- | --- | --- | --- | --- | --- | --- | --- |
| **1** | BraakStage | age | BraakStage | BrainNetStage | age | CAAType | BraakStage |
| **2** | BrainNetStage | CAATotalSev | BrainNetStage | BraakStage | BraakStage | brain weight | BrainNetStage |
| **3** | CAAMeningeal | AbStageTypical | CAAMeningeal | MTSPETSA | BrainNetStage | age | CAAMeningeal |
| **4** | CAAParenc | brain weight | CAAParenc | TSAAny | CAATotalSev | BrainNetStage | CAAParenc |
| **5** | age | BraakStage | age | SubpialMesTemp | brain weight | MTSPETSA | age |
| **6** | ThalStage | BrainNetStage | ThalStage | ThalStage | CAAType | BraakStage | ThalStage |
| **7** | CAAType | CAAAreas | CAAType | age | CAAMeningeal | CAAAreas | CAAType |
| **8** | brain weight | SubpialBrainstem | brain weight | CAAType | CAAParenc | CAAHippocampus | brain weight |
| **9** | SubpialBrainstem | CAAType | CAATotalSev | TSATotal | SubpialBrainstem | SubpialBrainstem | SubpialBrainstem |
| **10** | CAAAreas | MTSPETSA | SubpialBrainstem | SubpialBrainstem | CAAAreas | CAAParietal | CAATotalSev |
| **11** | CAATotalSev | BSSPETSA | CAAAreas | HippocTauStage | ThalStage | ThalStage | CAAAreas |
| **12** | MTSPETSA | CAAMeningeal | MTSPETSA | SubcorticalStage | MTSPETSA | CAAFrontal | MTSPETSA |
| **13** | CAAHippocampus | TempMicroinf | CAAHippocampus | brain weight | BSSPETSA | CAATemp | CAAHippocampus |
| **14** | CAAParietal | CAAParenc | CAAParietal | BSSPETSA | CAAHippocampus | CAAOccipital | CAAParietal |
| **15** | CAAFrontal | ThalStage | CAAFrontal | CAAOccipital | CAAParietal | TSAAny | CAAFrontal |
| **16** | CAAOccipital | CAAHippocampus | BSSPETSA | CAAParietal | CAAOccipital | CAACerebellum | CAAOccipital |
| **17** | HippocTauStage | CAAOccipital | CAAOccipital | PARTall | CAAFrontal | CAAMeningeal | HippocTauStage |
| **18** | CAATemp | CAAParietal | HippocTauStage | CAAMeningeal | CAATemp | SubpialMesTemp | BSSPETSA |
| **19** | BSSPETSA | CAAFrontal | CAATemp | CAAHippocampus | TSAAny | CAAParenc | CAATemp |
| **20** | TSAAny | CAATemp | TSAAny | MicroinfarctStage | CAACerebellum | CAATotalSev | TSAAny |
| **21** | CAACerebellum | TSAAny | CAACerebellum | CAACerebellum | AbStageTypical | HippocTauStage | CAACerebellum |
| **22** | SubpialMesTemp | FrontalMicroin | SubpialMesTemp | CAAParenc | SubpialMesTemp | BSSPETSA | SubpialMesTemp |
| **23** | AbStageTypical | CAACerebellum | AbStageTypical | CAATotalSev | HippocTauStage | FrontalMicroin | AbStageTypical |
| **24** | TempMicroinf | SubpialMesTemp | TempMicroinf | CAAAreas | TempMicroinf | TempMicroinf | TempMicroinf |
| **25** | FrontalMicroin | HippocTauStage | FrontalMicroin | CxSPETSA | FrontalMicroin | PARTdefinite | FrontalMicroin |
| **26** | PARTdefinite | ArgyrGrains | PARTdefinite | PARTdefinite | ParMicrin | AbStageTypical | PARTdefinite |
| **27** | ParMicrin | ParMicrin | ParMicrin | ArgyrGrains | PARTdefinite | ParMicrin | ParMicrin |
| **28** | ArgyrGrains | PARTdefinite | ArgyrGrains | TempMicroinf | ArgyrGrains | OccipMicroing | ArgyrGrains |
| **29** | OccipMicroing | CxSPETSA | OccipMicroing | CorticalStage | OccipMicroing | ArgyrGrains | OccipMicroing |
| **30** | CxSPETSA | OccipMicroing | CxSPETSA | AbStageTypical | CxSPETSA | PARTall | CxSPETSA |
| **31** | TuftedAst | TuftedAst | TuftedAst | FrontalMicroin | TuftedAst | TuftedAst | TuftedAst |
| **32** | PARTall | PARTall | PARTall | OccipMicroing | PARTall | CxSPETSA | PARTall |
| **33** | MicroinfarctStage | CorticalStage | SubcorticalStage | CAATemp | SubcorticalStage | CorticalStage | MicroinfarctStae |
| **34** | TSATotal | TSATotal | MicroinfarctStage | ParMicrin | CorticalStage | SubcorticalStage | TSATotal |
| **35** | SubcorticalStage | SubcorticalStage | TSATotal | CAAFrontal | TSATotal | TSATotal | SubcorticalStage |
| **36** | CorticalStage | MicroinfarctStage | CorticalStage | TuftedAst | MicroinfarctStage | MicroinfarctStage | CorticalStage |

Supplementary Table 1. Ranking of the CFAS Dataset Features using Different Feature Selection Techniques Seven feature-ranking methods were presented: Chi-Square (CHI), Gain Ratio (GR), Information Gain (IG), ReliefF (RF), Symmetrical Uncertainty (SmyUn), Least Loss (L2) and Variable Analysis (Va).

**Supplementary Table 2 T-test and p-values for all non-standard and demographic features**

| **No.** | **Feature** | **T test** | **P-value** |
| --- | --- | --- | --- |
| 1 | Age | 3.132 | 0.00 |
| 2 | Brain weight | -3.741 | 0.001 |
| 3 | Virchow-Robin Space (VRS) Expansion | 0.607 | 0.547 |
| 4 | Gender | -0.842 | 0.404 |
| 5 | Lewy Bodies in Substantia Nigra | 0.328 | 0.744 |
| 6 | Neuronal Loss in Substantia Nigra | 0.478 | 0.635 |
| 7 | Neuronal Loss in Hippocampus | 0.541 | 0.591 |
| 8 | Tangles in Temporal Lobe | -1.046 | 0.301 |
| 9 | Parenchymal CAA in Frontal Lobe | -1.734 | 0.090 |
| 10 | Gliosis in Hippocampus | 0.644 | 0.523 |

Supplementary Table 2. T-test and p-values for all non-standard and demographic features. The result shows that there statistically significant differences in the values of non-standard features between false positives and false negatives

#

# Glossary List

| **Feature** | **Description** |
| --- | --- |
| **BraakStage** | Refers to Braak Neurofibrillary Tangle Stage (0-VI) (Braak, Alafuzoff et al. 2006). Braak stages:  (I/II) when neurofibrillary tangle involvement is limited to the transentorhinal region of the brain, stages  (III/IV) when involvement of limbic regions such as the hippocampus appears.  (V/VI) when there is extensive neocortical involvement. |
| **BrainNetStage** | Brain-Net Tau Stage (1-6) - (Brain-Net Europe protocol for tau pathology) |
| **Aged** | Age of an individual at death |
| **CAATotalSev** | Cerebral amyloid angiopathy (CAA) total severity is the scores for leptomeningeal and parenchymal amyloid, giving a score out of maximum of 24 for severity in cortical areas. |
| **CAAMeningeal** | Cerebral amyloid angiopathy (CAA) Severity Meningeal as for parenchymal the scores are out of 12 |
| **Brain-weight** | Brain weight of an individual after death |
| **CAAType:** | Cerebral amyloid angiopathy (CAA) type as defined by Thal, (1) are cases with capillary amyloid and (2) only in larger vessels, (0) no CAA |
| **ThalStage** | Thal Abeta stage- the new brain -Net-Stage for Abeta. This is a five-stage scheme. This is based on the detection of immunopositive amyloid in cortical and subcortical areas,   1. progressive deposition of amyloid in neocortex, 2. allocortex or limbic, 3. diencephalon/basal ganglia 4. brainstem/midbrain, 5. cerebellum |
| **MTSPETSA** | Thorn-Shaped Astrcytes (TSA) subpial/epen mesial temporal |
| **CAAAreas** | Number of brain areas examined that have cerebral amyloid angiopathy (CAA). The number of anatomical areas involved from all the areas in the sampling set to obtain a measure of the extent (number of areas out of 9 maximum). |
| **Subpialbrainstem** | Subpial tau neurites in brainstem/subcortical region |
| **CAAParenc** | Cerebral amyloid angiopathy (CAA) Severity score (Love 2014) Parenchymal-So in any area CAA can be 1, 2 or 3 |
| **TSATotal** | Thorn-Shaped Astrcytes (TSA), presence (1) or absence (0) TSA in any brain area |
| **TSAAny** | Thorn-Shaped Astrcytes (TSA), Total number of areas with TSA |
| **CAAParietal** | Cerebral amyloid angiopathy (CAA) is present or not in Parietal Cortex |
| **CAAHippocampus** | Cerebral amyloid angiopathy (CAA) is present or not in Hippocampus and (OTG) occipitotemporal gyrus |
| **CAAFrontal** | Cerebral amyloid angiopathy (CAA) is present or not in Frontal Cortex |
| **CAAOcciptal** | Cerebral amyloid angiopathy (CAA) is present or not in Occipital Cortex |
| **BSSPETSA** | Thorn-Shaped Astrcytes (TSA) subpial/epend brainstem |
| **CAACerebellum** | Cerebral amyloid angiopathy (CAA) is present or not in Cerebellum |
| **AbStageTypical** | (1) Thal typical, (2) atypical |
| **HippocTauStage** | Hippocampal Lace Tau Stage based on progression through the hippocampus |
| **SubcorticalStage** | Subcortical lacune stage- number of subcortical areas that have microinfarcts |
| **CorticalStage** | Cortical microinfarct stage- number of cortical areas that have microinfarcts |
| **SubpialMesTemp** | Subpial tau neurites in mesial temporal |
| **PARTall** | Primary age-related tauopathy (PART), (1) PARTall Abeta 0-2/tau I-IV, (2) Abeta 3+/tau I-IV |
| **MicroinfractStage** | Total microinfarct stage- number of total of areas that have microinfarcts |
| **ArgyGrains** | Argyrophilic grains |
| **OccipMicroing** | Occipital Microinfarct |
| **ParMicrin** | Parietal Microinfarct |
| **PARTdefinite** | Primary age-related tauopathy (PART), PART-definite is defined as no Aβ pathology (Thal stage 0) and Braak NFT stage I-IV and PART-possible as mild Aβ pathology (Thal stage I-II)/Braak NFT stage I-IV (Crary, Trojanowski et al. 2014). |
| **FrontalMicroin** | Frontal Microinfarct |
| **TempMicroinf** | Temporal Microinfarct |
| **CxSPETSA** | Thorn-Shaped Astrcytes (TSA) subpial/epend cortex |
| **CAATemp** | Cerebral amyloid angiopathy (CAA) is present or not in Temporal Cortex |
| **TuffedAst** | Tufted parencymal astrocytes any area |
